# Patient-Reported and Technology-Assisted Monitoring in Orthotic Management of Adolescents With Idiopathic Scoliosis: Scoping Review

**DOI:** 10.2196/88880

**Published:** 2026-08-10

**Authors:** Anna Mathew, Kamila Sykorova, Nenad Pavel, Marianne Bakke Johnsen, Minna Pikkarainen, Sadeeq Ali, Mette Fløystad Kvammen, Parisa Gazerani

**Affiliations:** 1 Department of Rehabilitation Science and Health Technology, Faculty of Health Sciences, OsloMet – Oslo Metropolitan University, Oslo, Norway; 2 Department of Product Design, Faculty of Technology, Art and Design, OsloMet – Oslo Metropolitan University, Kjeller, Oslo, Norway; 3 Prosthetics and Orthotics, Department of Rehabilitation Science and Health Technology, Faculty of Health Sciences, OsloMet – Oslo Metropolitan University, Oslo, Norway; 4 Department of Sociology and Social Work, University of Agder, Kristiansand, Agder, Norway; 5 Norwegian Spine and Back Pain Association, Oslo, Norway; 6 ADEPT Research Group, Department of Life Sciences and Health, Faculty of Health Sciences, OsloMet – Oslo Metropolitan University, Pilestredet 50, Oslo, 0167, Norway, 47 67 23 53 97

**Keywords:** braces, health technology, health information systems, orthotic devices, patient monitoring, physiologic, sensors, scoliosis, surveys and questionnaires, self report, wearable electronic devices

## Abstract

**Background:**

Orthotic treatment for adolescents diagnosed with idiopathic scoliosis is long-term and often associated with challenges related to treatment adherence. Traditional patient monitoring strategies include clinical visits combined with patient discussions, clinical examination reports, and the use of standard questionnaires. Sensor-assisted monitoring is explored to improve treatment outcomes, with technology influencing different aspects of the health care system.

**Objective:**

The scoping review aimed to map evidence based on 3 research questions (RQs), namely user experience and perception, monitoring tools, and technology-assisted approaches, including smart orthoses with sensors, during orthotic treatment for an adolescent patient (aged 10 to 17.11 y) diagnosed with idiopathic scoliosis.

**Methods:**

A systematic search was conducted in MEDLINE, Embase, PsycINFO, Cochrane Library, CINAHL, Web of Science, and Scopus as 2 complementary search components. The primary search targeted studies on smart orthoses, also called braces, and scoliosis, while the secondary search focused on user experiences with orthotic treatment to capture all 3 RQs. PRISMA-ScR (Preferred Reporting Items for Systematic Reviews and Meta-analyses extension for Scoping Reviews) guidelines and checklist supported searching, analyzing, and reporting the results systematically. Predefined inclusion and exclusion criteria were followed for screening. All articles published up to May 2024 were included. Moreover, 2 independent reviewers screened 2088 articles in 2 stages of screening. Data coding was conducted using the 3 RQs by data extraction and its synthesis.

**Results:**

A total of 88 articles met the inclusion criteria. Evidence from user experience studies highlighted discomfort, psychosocial burden, and demand for real-time feedback. Monitoring relied primarily on patient-reported outcome measures (PROMs) such as questionnaires and logbooks. Culturally adapting to patients’ diverse global needs helps capture richer and more candid user perspectives. Sensor-based systems used temperature, force, or pressure, electromyography, and motion sensors to track orthosis wear time, pressure distribution, activity, and posture. Two overarching monitoring themes emerged: subjective (patient-reported or user experience) and objective (technology-assisted or sensor-based) monitoring. Integrated approaches synthesize both.

**Conclusions:**

Subjective clinical tools and objective technologies help monitor orthosis wear of adolescent patients with idiopathic scoliosis. Co-design of a user-centered scoliosis management system for adolescents that integrates PROMs with measured sensor data could provide a multidimensional view of orthosis adherence along with psychosocial and clinical effectiveness. Future research will focus on user-centered care, with real-time integrated monitoring, to provide clinically meaningful feedback that motivates adolescents to achieve their treatment goals.

## Introduction

### Background

Scoliosis refers to the deformity of the spine, thorax, and trunk. The root cause of scoliosis can differ. About 80% of cases are classified broadly by etiology as idiopathic scoliosis [1,2]. The International Scientific Society on Scoliosis Orthopedic and Rehabilitation Treatment reports that idiopathic scoliosis is diagnosed in seemingly healthy children and tends to progress rapidly at puberty [3].

Nonsurgical treatment options for scoliosis management include observation, physiotherapeutic-specific exercises, special inpatient rehabilitation, and bracing [3]. Bracing combined with exercise is an effective conservative treatment approach for patients with adolescent idiopathic scoliosis [4]. Since the last decade, scoliosis orthoses or braces have been considered the gold standard for decreasing the progression rate of curves, based on the multicenter study “Bracing in Adolescent Idiopathic Scoliosis Trial” from the United States and Canada, which demonstrated that the success of orthotic treatment increases with higher wearing hours [5]. However, treating adolescents with prolonged unpredictable treatment outcomes is challenging [6,7]. The most affected are female individuals with a higher diagnosis rate than male individuals [1], and the rate of deformity is highest at the beginning of puberty, during adolescence [2].

The hours of orthosis wear stand as an integral component of the treatment [8]. These orthoses can be rigid or soft, varying in construction, correction principles, and clinical outcomes. Ali et al [9], in the systematic review, compare 20 different orthoses and highlight their differences. Greater curve correction is obtained with rigid orthoses, while soft orthoses can deliver correction, enhanced comfort, and engage the patient’s muscles [9].

The Cobb angle is the clinical measure that quantifies the degree of spinal deformity. At the same time, the Risser stage assesses skeletal maturity on a 0‐5 scale, using either a US or a European scale where 5 means complete maturity. In addition to these clinical approaches, patient-reported questionnaires support with valuable information on the patients’ emotional and psychological burden [10]. Different patient monitoring tools support clinicians in long-term scoliosis management in adolescents by assessing the effectiveness of orthoses and enabling patient-focused treatment adjustments [11,12]. Digital health care–based monitoring using sensors can support the treatment journey [2]. As technology advances, there is a need to update and integrate sensors with digitalized monitoring solutions to improve bracing outcomes. Mobile-based health monitoring systems are emerging to track adolescent treatment compliance and well-being [13]. Scoliosis care and treatment protocols have been inconsistent globally, with regional variants. Different health care providers involved in scoliosis treatment identified the need for increased research collaboration and standardization of treatment protocols globally [2]. Accepting these diverse and significant contexts that address user needs within scoliosis, integrating both health-oriented and technology-driven strategies, can be useful for future co-designs.

### Context and Significance

#### User Experience and Perception

In scoliosis orthotic management, patient adherence to treatment goals is significant but challenging [8,10]. Adherence to orthotic treatment is a process that may begin with experiencing symptoms, consulting a therapist, becoming aware of visible spinal deformity, and receiving attention from family members. It may then progress to seeking care by accepting the diagnosis, discussing options with an orthotist, agreeing to treatment and living with an orthosis, and adapting psychosocially. Each step can influence the patient’s compliance. Thus, treatment with orthosis is a process that depends on the combined effort of patients, family, physicians, and orthotists to reach treatment goals [14,15]. The treatment aims to enhance the patient’s quality of life by addressing current and long-term needs. The target of scoliosis management includes preventing curve progression and thereby reducing physical disability, improving body aesthetics, delivering daily functioning, overall well-being, including mental health, and improving self-esteem [16]. To achieve these broader treatment goals, it is essential to incorporate evaluation and monitoring methods that capture patients’ experiences and perceptions of treatment challenges and their preferences [17].

#### Monitoring Tools

Different questionnaires are designed for monitoring patients. Questionnaires are frequently used to objectively assess patients’ subjective experiences, such as physical function and psychological well-being [18-20]. Capturing meaningful patient-reported information enables health personnel to understand treatment adherence better and recommend more effective interventions. Clinicians need to discuss essential aspects of orthosis adherence, such as the actual hours the orthosis is worn compared with the prescribed duration, and the effectiveness of prescribed exercises [21]. This information can support prognosis and guide treatment decisions. Record cards or patient logbooks can give additional information to track patient observations between visits [22]. These methods represent traditional and nontechnological approaches to monitoring. While pain is not typically a primary clinical issue in orthosis-treated adolescent patients with idiopathic scoliosis, some questionnaires include pain subscales because these tools were originally developed for broader scoliosis populations. Pain-related items were therefore reported when present in included studies but interpreted cautiously.

#### Technology-Assisted Approaches

Sensor-assisted technologies are evolving toward personalized and intelligent orthotic management systems that enable real-time monitoring and communication among patients, caregivers, and health care professionals. Various wearable sensors have been developed to objectively monitor orthosis use, including temperature, pressure, force, and motion sensors (eg, studies by Fregna et al [8], Rahman et al [23], Zou et al [24], Cordani et al [25], Tymińska et al [26], and Donzelli et al [27]). For instance, in the study by Donzelli et al [27], sensor data provides information on actual orthosis wear hours, aiding clinicians in planning personalized treatment goals. Smart textile sensor systems for scoliosis monitoring are attracting research interest [28]. These technologies can quantify orthosis wear time and adherence, providing clinicians with objective information to support individualized treatment decisions. Data collected from these devices can be displayed through mobile applications or web-based platforms, allowing continuous monitoring of treatment progress [17]. More recently, wearable sensors integrated with AI have expanded opportunities for personalized monitoring and decision support (eg, studies by Cordani et al [25], Dehzangi et al [29], Zhu et al [30], and Li et al [31]). However, further evidence is needed to determine how these technologies can best support clinical decision-making and improve outcomes in adolescents.

Among existing reviews of adolescent idiopathic scoliosis management, the study by Raudenbush et al [32] provides an overview of studies evaluating, diagnosing, and treating adolescent patients with idiopathic scoliosis. Previous scoping reviews have explored evidence of successful treatment outcomes predominantly using the Cobb angle and quality-of-life questionnaires [33], and another study consolidated user needs, particularly regarding body image schema in adolescent patients with idiopathic scoliosis [34]. The scoping review includes studies from 2020 that capture the themes relevant to integrated sensor-based wearables, particularly electromyography systems, AI, and relevant platforms, and reports the persistent gap in laboratory design and long-term clinical validation [35]. There appears to be a lack of scoping review that integrates patient-reported outcome measures (PROMs) with technology-assisted monitoring approaches for adolescent idiopathic scoliosis.

It is important to distinguish between subjective and objective approaches to monitoring orthotic treatment in adolescents with scoliosis. Subjective approaches capture the patient’s lived experience and perceptions, including comfort, usability, adherence challenges, psychosocial burden, and satisfaction with treatment, typically assessed through PROMs, questionnaires, or interviews. In contrast, objective approaches rely on technology-assisted monitoring tools, such as embedded sensors, temperature or pressure measurements, wear-time trackers, or other digital systems that quantify orthosis use or biomechanical parameters without relying on self-report. A third category includes integrated approaches, which combine patient-reported measures with objective sensor-based data to provide a more comprehensive understanding of both adherence and user experience. This distinction provides a clearer conceptual framework for synthesizing the existing literature and interpreting how different monitoring strategies contribute to treatment optimization.

### Aim and Research Questions

The primary aim of this study was to determine the existing evidence on monitoring approaches spanning user experiences, clinical patient-monitoring tools, and technology-assisted systems for optimizing adherence and outcomes in the treatment and management of adolescent idiopathic scoliosis.

The integration of health and technology requires a detailed review to identify gaps and emerging technologies with the potential to improve patient care, especially for adolescents. This scoping review aims to estimate current evidence on user experience and perception of adolescents on diverse monitoring tools, including the technological and clinically assisted approaches, as part of conservative orthotic management for adolescents with idiopathic scoliosis. The research questions (RQs) are listed in Textbox 1.

Textbox 1. **Three research questions (RQs).**

**RQ-A**

- User experience and perception: What is known about user experience, perception, and needs related to orthosis-related monitoring in adolescents with idiopathic scoliosis?

**RQ-B**

- Monitoring tools: What patient-reported or digital monitoring tools have been used to assess adherence, comfort, or treatment progress in bracing among adolescents with idiopathic scoliosis?

**RQ-C**

- Technology-assisted approaches: What technology-assisted or sensor-based monitoring systems have been used in the management of bracing for adolescents with idiopathic scoliosis and which parameters do they capture?

## Methods

### Rationale for Scoping Review

A systematic review addresses defined, narrow questions with a strict assessment of evidence, while scoping reviews are ideal for examining emerging areas, clarifying definitions, and identifying research gaps [36,37]. A scoping review is particularly well-suited to this topic, given the complexity of the RQs and the interdisciplinary nature, allowing exploration of the breadth and depth of available evidence and supporting the identification of key concepts and gaps. Given the diverse study designs on this topic, including both health and technological perspectives, a scoping review enables a more inclusive and exploratory synthesis of available knowledge.

### Framework

Arksey and O’Malley [37] provide the methodological framework of the review process that included systematic identification, selection of articles based on inclusion and exclusion, and charting of evidence across predefined RQs [38]. Data extraction into tables, its analysis, and presentation were defined by the Joanna Briggs Institute that uses qualitative content analysis and data visualization approaches [39]. The PRISMA-ScR (Preferred Reporting Items for Systematic Reviews and Meta-Analyses extension for Scoping Reviews) and the checklists were used to report these processes as suggested by [40]. In the review, the PRISMA-ScR guidelines and the PRISMA-ScR checklist were followed. CONSORT-EHEALTH (Consolidated Standards of Reporting Trials of Electronic and Mobile Health Applications and Online Telehealth) was reviewed for completeness because some included studies involve tech-assisted interventions, but PRISMA-ScR remains the main reporting framework [41].

### Protocol and Registration

The protocol was prepared before conducting the search. RQs helped to guide the database search and the data collection in accordance with the scoping review framework. It is registered in INPLASY (ID INPLASY2025100032).

### Information Sources and Search

The research team developed the scoping review protocol, drawing on existing literature to initiate discussions with university librarians, after a joint dialogue with specialized librarians and researchers. Search trials of the Medline database were prepared and tested first. A 2-step search strategy was finalized to comprehensively address all RQs. Based on this defined search strategy, librarians conducted the search in the 7 databases, namely MEDLINE, Embase, PsycINFO, Cochrane Library, CINAHL, Web of Science, and Scopus [42,43]. The results from both searches in each database were consolidated to generate the final single EndNote 21 (Clarivate) file that was compiled by the librarians. All articles published up to May 2024 were considered. Both the search strategies for each database are provided in Multimedia Appendix 1.

### Eligibility Criteria

The primary criterion is that every article must include at least 1 patient participant, aged 10 to 17 years and 11 months (17.11 y) and diagnosed with idiopathic scoliosis [3]. If studies contain diagnoses other than adolescent idiopathic scoliosis and results are presented together, the article shall be excluded. Textbox 2 illustrates inclusion and exclusion criteria based on the Population, Intervention, Comparison, Outcome, and Study design (PICOS) framework, along with other generic criteria.

Textbox 2. **Inclusion and exclusion criteria.**

**Inclusion Criteria**

- Population
  (1) Diagnosis: Patients diagnosed with adolescent idiopathic scoliosis
  (2) Age: Between 10 years and 17.11 years.
- Intervention: Idiopathic scoliosis treatments with orthosis, with or without physical activities (including training, physical activity, and exercises).
- Comparators: All comparators are eligible (including no comparator, placebo, or other orthotic treatments).
- Outcomes: Not applicable (scoping review design).
- Study: All articles meeting search criteria considered.
- Publication type: Peer-reviewed articles and conference papers; opinions and medical hypotheses.
- Availability: Full-text accessible through institutional affiliations (eg, OsloMet, Aalborg University).
- Language: English only.

**Exclusion Criteria**

- Population
  (1) Diagnosis:
  Congenital scoliosis, neuromuscular scoliosis, or other nonadolescent idiopathic scoliosis types.
  Articles with incomplete participant information or without patient participants.
  Studies with healthy subjects.
  (2) Age: Below 10 years or above 17.11 years and unclear age information from full text.
- Intervention: Nonorthotic treatments, including surgical interventions.
- Study: Simulation studies without patient involvement (eg, model-based or radiograph-only analysis). Studies focused solely on orthosis or prototype development without clinical or patient testing, without patient-reported outcomes or user perspectives.
- Publication type: Book reviews, editorials, conference abstracts (eg, oral presentations, short abstracts without details), short papers without peer review, commentaries, errata, letters to the editor.
- Availability: Articles not accessible in a readable format; ongoing or incomplete trials without published results.
- Language: Articles not available in English and with English abstracts but foreign-language titles or published in non-English journals.

### Selection of Sources of Evidence

#### Overview

The Rayyan software tool was used to organize and manage the screening process. Each article was compared against its duplicates to retain only 1 version, following priority order based on the following database sources: MEDLINE, Embase, PsycINFO, Cochrane Library, CINAHL, Web of Science, and Scopus. Rayyan’s reference comparison features helped highlight differences between duplicate entries. Duplicates were manually removed to ensure accuracy, despite the ability of Rayyan’s feature to remove duplicates as groups with a certain percentage of duplication.

Articles published in health sciences typically present patient information clearly, particularly in the Methods section. However, this level of detail was often lacking in technology-focused or prototype-based studies, even when participants were involved. When further clarification was required, articles were annotated using the labeling feature of Rayyan. Such articles with partial or ambiguous information were retained and moved to full-text screening. Through digital meetings, the 2 independent researchers (AM and PG) resolved ambiguity according to the predefined inclusion and exclusion criteria. Most disagreements were resolved through discussions, and when necessary, a third researcher (MP) provided input. For example, disagreements regarding age limits in the eligibility criteria were resolved using the International Scientific Society on Scoliosis Orthopaedic and Rehabilitation Treatment (SOSORT) 2012 guidelines, which specify that adolescent idiopathic scoliosis is for ages 10 to 17 years (specifically 17 y and 11 mo), ages 3 to 9 years as juveniles, and ages 18 years and older as adults [3]. Furthermore, in cases where participant demographics varied from article to article, independent reviewers resolved them by verifying the full text during online meetings. Full texts were retrieved through university affiliations, specifically OsloMet and Aalborg University, and 22 articles were acquired with support from OsloMet University for full-text screening.

Language was considered as a criterion; articles that had English abstracts but were written in foreign languages fell outside the scope of this review. All articles excluded during the abstract and title screening phase used predefined exclusion labels in the Rayyan software. For example, the label “foreign language” was used to exclude articles. If the title appeared in square brackets (eg, [German] in the Rayyan software tool) or if the journal title was in a non-English language, they were excluded at title and abstract screening. The user-defined label in the Rayyan software helped trace the stage.

Participant demographics were another eligibility criterion. Articles that failed to meet the inclusion criteria, specifically age or disease diagnosis, were the most common and challenging eligibility criteria. If the article included at least 1 participant younger than 10 years or older than 17.11 years, it was excluded despite valuable insights. Articles with unclear age ranges but reporting mean and SD, or referencing Risser stages, were considered on a case-by-case basis. Studies that included both juvenile and adolescent participants were excluded as results cannot be separated (eg, study by Lou et al [44]).

#### Data Charting

During the charting process, independent reviewers encountered challenges organizing multidisciplinary articles, which were resolved by iteratively refining a table structure based on charted data samples. The final table was refined through multiple meetings. The extracted data items were organized as columns.

#### Data Items

During data extraction, included studies were categorized according to the primary monitoring approach used: (1) subjective monitoring, focusing on patient-reported outcomes and user experience; (2) objective monitoring, using technology-assisted or sensor-based tools to quantify orthosis use or treatment parameters; or (3) integrated monitoring, combining both subjective and objective measures. This categorization was applied consistently across study characteristics and informed the narrative synthesis.

The data extraction table included columns for study characteristics, including participant demographics related to scoliosis and age, followed by columns for clinical and technological RQs. Due to variability in how age was reported across studies, this information was standardized as “mean age (SD)” supplemented with reported age ranges (lower-upper limits) in Multimedia Appendix 2. These age data are finalized for each selected article by verifying inclusion criteria, demographic tables, or results sections, and are presented as age range (minimum year-maximum year). For example, when the Risser stage is represented as the upper limit, it is presented as range (minimum year-maximum Risser 2/3). Risser stages 2 to 4 correspond to mean ages ranging from 13.1 (SD 1.1) to 14.8 (SD 1.0) years in adolescent patients with idiopathic scoliosis, which is less than 18 years [45]. Other study data extracted as part of this review included publication year, country data, author information, journal data, participant data, including the male-to-female ratio, and study methodology [39]. In alignment with the RQs, additional data were extracted on orthosis types, clinical monitoring instruments (eg, questionnaires), technology-based sensor monitoring parameters, data analysis methods, participant feedback and their perspectives, study strengths, results, and proposed future directions. The result of this phase is a detailed data extraction table, which is provided in Multimedia Appendix 2.

#### Data Synthesis Approach

The refined table aligns with the 3 RQs, helping condense the 2 main themes that emerged from data synthesis. AI was not used in data synthesis. The detailed data extraction table records key findings from each article. The time-based evolution of selected data items based on the 3 RQs is presented as illustrative figures that are created using these data in Microsoft Excel using Insert Tab/PivotChart and PivotTable features.

### Ethical Considerations

The research was conducted independently. The funder (University) played no role in the formulation of the study design, data analysis and interpretation, or writing of this paper.

## Results

### Overview

A total of 5470 records were identified from 7 databases: MEDLINE (n=1016), Embase (n=1472), PsycINFO (n=30), Cochrane Library (n=178), CINAHL (n=518), Web of Science (n=798), and Scopus (n=1458). Due to overlap between the 2 search strategies, approximately 61.8% (3382/5470) of the duplicate articles were removed. A total of 2088 articles were identified for further screening based on their titles and abstracts, followed by full-text screening. Exclusion of articles was applied based on the predefined eligibility criteria in Textbox 2, specifically targeting participants diagnosed with idiopathic scoliosis and aged 10-17.11 years.

**Figure 1.**
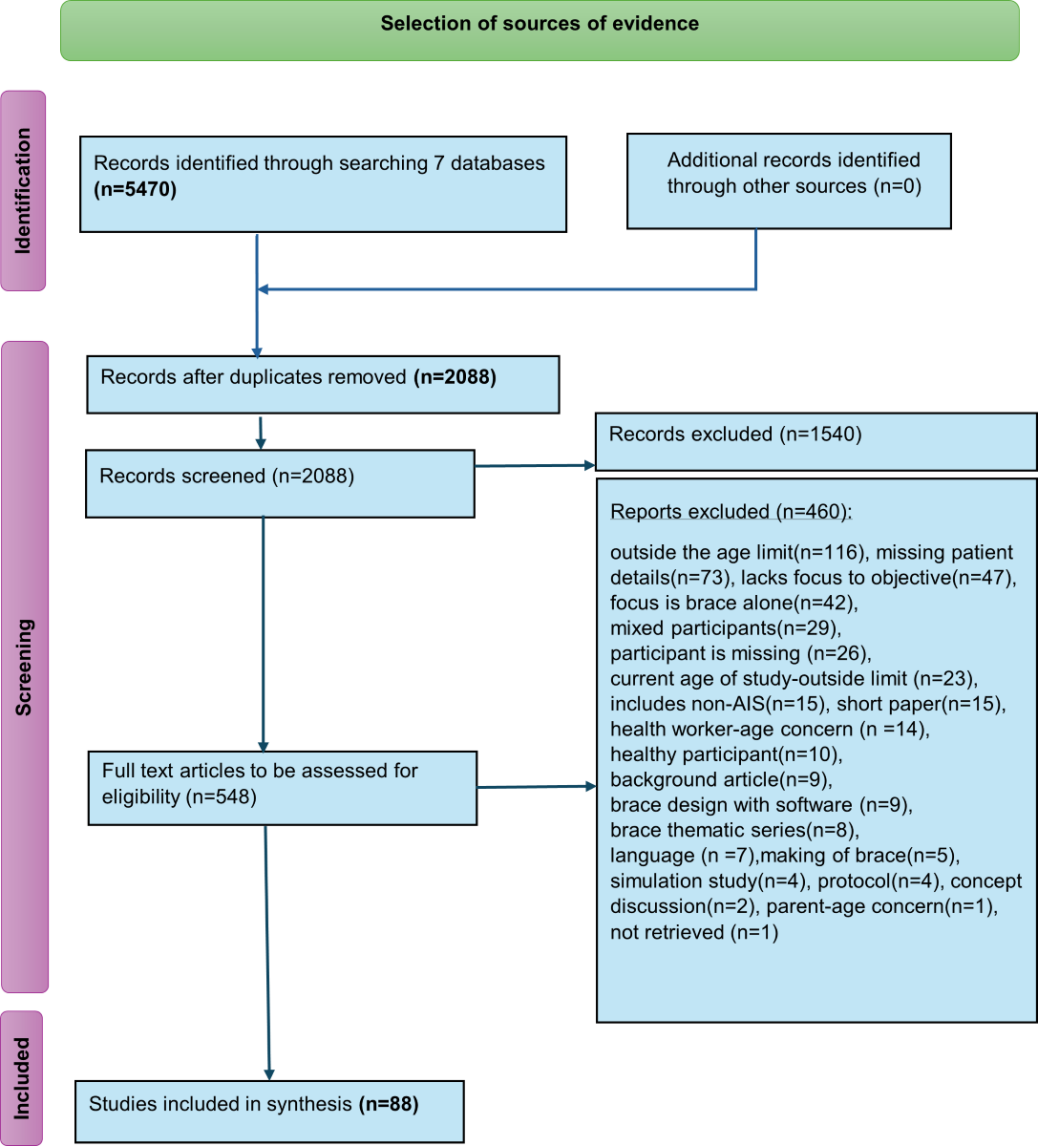
Flow diagram based on PRISMA-ScR (Preferred Reporting Items for Systematic Reviews and Meta-analyses extension for scoping reviews).

During full-text screening, articles (n=460) were excluded using user-defined exclusion criteria (as illustrated in Figure 1), which helped identify the stage at which each article was removed in the Rayyan software. For example, 7 articles were excluded at the full-text stage that were not captured at the first phase and were labeled as “language (n=7)” as in Figure 1. Article exclusions were predominantly based on participant demographics and alignment with the review objectives. Some exclusion categories were conceptually related. To avoid duplicate counting, each article was assigned 1 primary reason that was the most appropriate and explanatory reason. Exclusion reasons include age outside the inclusion criteria (n=116), follow-up study design with older participants (n=23), missing patient or participant information (n=73 and n=26), protocol-only publications without participants (n=4), and short papers with missing data (n=15). Studies with ineligible participants were excluded, for instance, studies involving surgical interventions (considered mixed participants; n=29), nonidiopathic scoliosis diagnoses (n=15), healthy participants (n=10), and parents (n=1). Delphi studies involving clinicians only were also excluded (n=14). The remaining exclusions were due to a lack of alignment with the scoping review objectives, including orthosis-focused, design-, simulation-, manufacturing-, thematic-, background-, or concept-based studies. Moreover, 1 article was excluded because it was not available in a readable format. A total of 88 studies (Table 1) were included for data extraction using Microsoft Excel. Each row in the data extraction table corresponds to an article, thereby yielding 88 rows. Table 1 summarizes the study characteristics of each included article based on the research objectives. This process is presented in Figure 1 as a flow diagram in accordance with PRISMA-ScR guidelines published by Tricco et al in 2018 [40].

**Table 1.** Summary of results.

Research questions	Title of article	Study demographics (Adolescent participants with idiopathic scoliosis)					Themes: subjective (S), objective (O), and integrated (I)		
		Year	Country	Female	Male	Age range (y): (Minimum -Maximum)	Report on orthosis (Brace)	Monitoring tool	S vs O vs I
*RQ^a^-A only (Subjective*)									
A1	Adolescents’ Experience during Brace Treatment for Scoliosis: A Qualitative Study [46]	2022	China	13	2	10-16	Type missing (report brace)	Interview	S
*RQ-B only (Subjective*)									
B1	Chinese Adaptation of the Bad Sobernheim Stress Questionnaire for Patients With Adolescent Idiopathic Scoliosis Under Brace Treatment [47]	2015	China	71	15	10-16	Brace (Shanghai Delin Artificial Limb Recovering Equipment, China)	BSSQ^b^ (Deformity and -Brace), SRS-22^c^	S
B2	Cross-Cultural Adaptation and Validation of the Bad Sobernheim Stress Questionnaire in Iranian Adolescents with Idiopathic Scoliosis Using Thoracolumbar Orthoses [48]	2022	Iran	40	15	12-17	TLSO^d^	BSSQ-Brace, SRS-22r	S
B3	Greek Adaptation and Validation of the Bad Sobernheim Stress Questionnaire-Brace and the Bad Sobernheim Stress Questionnaire-Deformity [49]	2023	Greece	46	1	11-17	Asymmetric Scoliosis Brace by SLC	BSSQ (Deformity and -Brace), SRS-22	S
B4	Persian adaptation of the Bad Sobernheim stress questionnaire for adolescent with idiopathic scoliosis [50]	2020	Iran	38	15	10-16	Milwaukee brace	BSSQ (Deformity and Brace), SRS-22	S
B5	Polish adaptation of Bad Sobernheim Stress Questionnaire-Brace and Bad Sobernheim Stress Questionnaire-Deformity [51]	2009	Poland	35	0	12-16	Cheneau brace	BSSQ (Deformity and -Brace), SRS-22	S
B6	Spanish validation of Bad Sobernheim Stress Questionnaire (BSSQ (brace).es) for adolescents with braces [52]	2010	Spain	33	2	10-16	Rigo System Cheneau Brace	BSSQ-Brace, SRS-22	S
B7	The Turkish version of the Brace Questionnaire in brace-treated adolescents with idiopathic scoliosis [53]	2018	Turkey	25	3	10-17	TLSO	BrQ^e^, SRS-22, BSSQ-Brace	S
B8	Validation of a Korean version of the quality-of-life profile for spine deformities (QLPSD) in patients with adolescent idiopathic scoliosis [54]	2022	South Korea	91	13	10.4-17.1	Type missing (report brace)	QLPSD, SRS-22	S
B9	Validation of the Italian spine youth quality of life (ISYQOL) in Korean population [55]	2021	South Korea	120	0	10.4-17.1	Type missing (report brace)	ISYQOL, SRS-22	S
B10	Validation of the Korean version of the Brace Questionnaire [56]	2018	South Korea	93	10	10-16	TLSO	K-BrQ^f^, K-SRS-22^g^	S
B11	Revisiting the psychometric properties of the Scoliosis Research Society-22 (SRS-22) French version [57]	2017	Canada	307	45	10-17	Type missing (report brace)	SRS-22fv	S
B12	Whether Orthotic Management and Exercise are Equally Effective to the Patients with Adolescent Idiopathic Scoliosis in Mainland China? [58]	2018	China	19	5	11-14	Rigid TLSO	SRS 22	S
B13	The relationship between quality of life and compliance to a brace protocol in adolescents with idiopathic scoliosis: a comparative study [59]	2009	South Africa	31	0	13-16	Rigo System Cheneau Brace	BrQ	S
B14	The influence of brace on quality of life of adolescents with idiopathic scoliosis [60]	2006	Greece	32	4	12-17	Boston brace	BrQ	S
B15	The importance of trunk perception during brace treatment in moderate juvenile idiopathic scoliosis: What is the impact on self-image? [61]	2017	Italy	54	0	10-17	Cheneau, Boston, and Milwaukee	TAPS^h^, SRS-22	S
B16	The efficacy of Schroth exercises combined with the Cheneau brace for the treatment of adolescent idiopathic scoliosis: a retrospective controlled study [62]	2022	China	157	NR	10-16	Cheneau-type TLSO	SRS-22r, EQ-5D	S
B17	The effect of Schroth exercises added to the standard of care on the quality of life and muscle endurance in adolescents with idiopathic scoliosis-an assessor and statistician blinded randomized controlled trial: “SOSORT^i^ 2015 Award Winner” [63]	2015	Canada	47	3	12.7-14.2	TLSO, Charleston, Providence Combination of Providence/TLSO brace	SEQ^j^, SAQ^k^, SRS-22r	S
B18	The effect of compliance to a Rigo System Cheneau brace and a specific exercise program on idiopathic scoliosis curvature: A comparative study: SOSORT 2014 award winner [64]	2014	South Africa	80	0	12-16	Rigo System Cheneau (RSC) brace	BrQ, HSPQ^l^, 16PF^m^	S
B19	The Effect of Bracing on Spinopelvic Rotation and Psychosocial Parameters in Adolescents with Idiopathic Scoliosis [65]	2019	Iran	28	2	10-Risser 2	Milwaukee, TLSO	Persian BSSQ and BrQ	S
B20	Quality of life and patient satisfaction in bracing treatment of adolescent idiopathic scoliosis [66]	2018	Argentina	43	0	10.8-14.5	TLSO	BrQ	S
B21	Quality of life after conservative treatment of adolescent idiopathic scoliosis [67]	2008	Greece	32	0	12-16	modified Boston brace	BrQ	S
B22	Psychological Effects of the SRS-22 on Girls With Adolescent Idiopathic Scoliosis [68]	2018	United States	45	0	10-17	Type missing (report braced)	BAS^n^, SRS-22	S
B23	Patients’ and Parents’ Perceptions of Appearance in Scoliosis Treated with a Brace: A Cross-Sectional Analysis [69]	2014	Poland	41	0	10-17	Cheneau brace	SAQ-pl	S
B24	Novel questionnaire to enhance brace wear adherence in patients with adolescent idiopathic scoliosis and the relationship of the quality of life [22]	2021	Thailand	35	5	11-17.3	Type missing (report braced)	Brace questionnaire	S
B25	Mindset correlates with health-related quality of life assessment in patients with adolescent idiopathic scoliosis [70]	2021	United States	94	16	10-Risser 2	TLSO	SRS-30 HRQoL^o^ survey and Health Mindset Scale	S
B26	Longer Brace Duration Is Associated with Lower Stress Levels and Better Quality of Life in Adolescents with Idiopathic Scoliosis [71]	2023	Italy	38	8	11-16	Sforzesco, Chêneau	BSSQ, ISYQoL	S
B27	Is higher compliance to brace therapy associated with poorer quality of life and self-image? A 36-months follow-up study [72]	2023	Italy	61	7	10-16	TLSO rigid brace	SRS-22r	S
B28	Flexibility Predicts Curve Progression in Providence Nighttime Bracing of Patients With Adolescent Idiopathic Scoliosis [73]	2016	Denmark	60	3	10-16	Providence brace	Danish version SRS-22	S
B29	Female patients’ and parents’ assessment of deformity- and brace-related stress in the conservative treatment of adolescent idiopathic scoliosis [74]	2012	Poland	63	0	10-17	Cheneau brace	Polish BSSQ (Deformity and Brace)	S
B30	Exploring mass customization and textile application in medical products: re-designing scoliosis brace for shorter production lead time and better quality of life [75]	2020	China	10	0	11-15	Garment brace & hard brace	BrQ questionnaire	S
B31	Enhancing Idiopathic Scoliosis Patients’ Outcomes Through Functional Rehabilitation Training in Combination with Orthosis [76]	2023	China	30	64	10-17	Fabricated orthosis	SF-36^p^	S
B32	Effect of different undergarment designs on the compliance and acceptance of the patients with adolescent idiopathic scoliosis under orthotic treatment [77]	2022	China	37	1	10-15	Spinal orthosis	SRS-22r and Brace Questionnaire (BrQ)	S
B33	Effect of bracing on the quality of life of adolescents with idiopathic scoliosis [78]	2004	United States	173	41	10.1-17.7	Type missing (report braced)	CHQ PF-28^q^, PODCI^r^	S
B34	Differences in deformity and bracing-related stress between rural and urban area patients with adolescent idiopathic scoliosis treated with a Cheneau brace [79]	2011	Poland	64	0	10-17	Cheneau brace	Polish BSSQ-brace and BSSQ deformity	S
B35	Core stabilization exercises versus scoliosis-specific exercises in moderate idiopathic scoliosis treatment [80]	2019	Turkey	30	0	12-Risser 3	Type missing (report braced)	WRVAS^s^, POTSI^t^, SRS-22	S
B36	Body-Related Attentional Bias in Adolescents Affected by Idiopathic Scoliosis [81]	2023	Italy	14	0	12-16	Type missing (report brace treatment)	SRS-22r, Visual Match-to-Sample Task	S
B37	Body Image and Quality-of-Life in Untreated Versus Brace-Treated Females With Adolescent Idiopathic Scoliosis [82]	2016	United States	319	0	10-15	Type missing (report brace)	SAQ, PedsQoL^u^	S
B38	Back Pain Prevalence Is Associated With Curve-type and Severity in Adolescents With Idiopathic Scoliosis: A Cross-sectional Study [83]	2017	Australia	158	23	10-17	Type missing (report brace wearing)	RMDQ^v^, BPI^w^	S
B39	An alternative to a randomized control design for assessing the efficacy and effectiveness of bracing in adolescent idiopathic scoliosis [84]	2015	China	63	5	10-15	TLSO	SRS-22, STAI^x^, BDI-II^y^	S
*RQ-C only (Objective*)									
C1	A compliance real-time monitoring system for the management of the brace usage in adolescent idiopathic scoliosis patients: a pilot study [30]	2021	China	23	5	10-15	Chêneau brace	Force	O
C2	Analysis of the corrective forces exerted by a dynamic derotation brace (DDB) [85]	2011	Greece	38	6	10-17.2	Dynamic derotation brace (DDB)	Force	O
C3	A smart point-of-care compliance monitoring solution for brace treatment of adolescent idiopathic scoliosis patients [29]	2021	United States	3	0	10-13	TLSO	Force and motion	O
C4	A wireless sensor network system to determine biomechanics of spinal braces during daily living [86]	2010	Canada	5	1	10-13	TLSO	Force	O
C5	Biomechanical Assessment of Providence Nighttime Brace for the Treatment of Adolescent Idiopathic Scoliosis [87]	2016	Canada	16	2	10-16	Providence brace	Pressure	O
C6	Biomechanical evaluation of the Milwaukee brace [88]	1998	China	9	0	10-14.8	Milwaukee brace	Strap pressure	O
C7	Boston brace correction in idiopathic scoliosis: a biomechanical study [89]	2003	United States	12	0	13-14	Boston brace	Pressure matrix	O
C8	Compliance with night-time overcorrection bracing in adolescent idiopathic scoliosis: Result from a cohort follow-up [90]	2020	France	19	1	11.2-15.6	“CAEN^z^” brace	Temperature	O
C9	Correlation between quantity and quality of orthosis wear and treatment outcomes in adolescent idiopathic scoliosis [91]	2004	Canada	12	3	12.2-16.8	Boston brace	Force	O
C10	Early intervention in nonoperative management of adolescent idiopathic scoliosis [92]	2000	China	9	0	10.5-14.0	TLSO (underarm brace)	Pressure and tension	O
C11	Effectiveness and biomechanics of spinal orthoses in the treatment of adolescent idiopathic scoliosis (AIS) [93]	2000	China	33	0	10-15	TLSO	Pressure and tension	O
C12	Effect of Compliance Counseling on Brace Use and Success in Patients with Adolescent Idiopathic Scoliosis [94]	2016	United States	154	17	10.2-16.0	TLSO	Temperature	O
C13	Electromyography of scoliotic patients treated with a brace [95]	2003	Canada	11	0	12-16	Boston brace	EMG^aa^	O
C14	Electronic monitoring of scoliosis brace wear compliance [23]	2010	United States	8	2	10.3-16.4	Wilmington scoliosis brace (TLSO)	Temperature	O
C15	Electronic monitoring of orthopedic brace compliance [96]	2015	United States	52	3	10-Risser 2	Wilmington brace	Temperature	O
C16	Forces exerted during exercises by patients with adolescent idiopathic scoliosis wearing fiberglass braces [97]	2006	Italy	14	3	12-17	Fiberglass braces	Pressure	O
C17	How quantity and quality of brace wear affect the brace treatment outcomes for AIS [98]	2016	Canada	52	8	10-Risser 2	TLSO	Force	O
C18	Load compliance monitor system for the treatment of scoliosis [99]	1999	Canada	1	0	13	Boston brace	Force	O
C19	Mechanical and Clinical Evaluation of a Shape Memory Alloy and Conventional Struts in a Flexible Scoliotic Brace [100]	2018	China	2	0	10-14	Garment brace	Pressure	O
C20	Objective compliance of adolescent girls with idiopathic scoliosis in a dynamic SpineCor brace [101]	2010	Switzerland	12	0	10-15.6	Dynamic SpineCor brace	Temperature	O
C21	Outcomes for nighttime bracing in adolescent idiopathic scoliosis based on brace wear adherence [102]	2024	United States	103	19	10-16	Providence nighttime brace	Temperature	O
C22	Relationships between strap tension, interface pressures and spine correction in brace treatment of scoliosis [103]	2002	Canada	35	0	10-16	Boston brace	Pressure from force	O
C23	Study of the pressures applied by a Cheneau brace for correction of adolescent idiopathic scoliosis [104]	2008	France	30	2	10-Risser 2	Cheneau brace	Pressure	O
C24	The association between brace compliance and outcome for patients with idiopathic scoliosis [105]	2005	United States	30	4	10-16	Wilmington jacket	Temperature	O
C25	The biomechanical effectiveness of the Boston brace in the management of adolescent idiopathic scoliosis [106]	1989	United Kingdom	12	2	11-16	Boston brace	Pressure and strap force	O
C26	The effect of time on qualitative compliance in brace treatment for AIS [107]	2008	Canada	0	11	11-14.9	Boston brace	Pressure from force	O
C27	The Influence of Body Habitus on Documented Brace Wear and Progression in Adolescents With Idiopathic Scoliosis [108]	2020	United States	159	16	10-15.8	Boston brace, TLSO	Temperature	O
C28	Validation of a miniature thermochron for monitoring thoracolumbosacral orthosis wear time [109]	2012	United States	7	0	13.3-15.7	TLSO	Temperature	O
C29	Effect of the application of the Boston brace system on the electrical activity of the paravertebral muscles in adolescents with idiopathic scoliosis: preliminary report [110]	1982	Canada	6	0	11-16	Boston brace	EMG	O
C30	Lumbopelvic postural differences in adolescent idiopathic scoliosis: A pilot study [111]	2022	United States	21	6	11-17	Type missing (report braced)	EMG	O
C31	Is daily walking distance affected in adolescent idiopathic scoliosis? An original prospective study using the pedometer on smartphones [112]	2020	France	18	1	11-17	Night time brace	Motion	O
RQ-A ∩ RQ-B (Subjective)									
AB1	What factor induces stress in patients with AIS under brace treatment? Analysis of a specific factor using exploratory factor analysis [113]	2021	Japan	66	3	10-16	Underarm brace or Boston brace	BSSQ, SRS-22	S
AB2	The experience of brace treatment in children/adolescents with scoliosis [114]	2006	Greece	9	3	10-16	Type missing (report braced >12 h)	Interview	S
AB3	The effect of rigid versus flexible spinal orthosis on the clinical efficacy and acceptance of the patients with adolescent idiopathic scoliosis [115]	2008	China	Not reported out of 43	Not reported out of 43	10-14	SpineCor and rigid spinal orthosis	VAS^ab^, 4 open Qn	S
AB4	Motivations for Compliance With Bracing in Adolescent Idiopathic Scoliosis [116]	2017	United States	39	0	11-15	Boston brace	Scoliosis Compliance Questionnaire	S
AB5	Psychosocial adaptation to wearing the Milwaukee brace for scoliosis. A pilot study of adolescent females and their mothers [117]	1984	United States	16	0	14-17	Milwaukee brace	Interview	S
AB6	Is self-image, in reference to the gravitational vertical, altered in adolescent idiopathic scoliosis? A multicenter, single-blind, case-control study [118]	2022	France	0	63	12.20-14.84	Type missing (report braced)	SRS-22, WRVAS, SAQ, TAPS	S
RQ-B ∩ RQ-C (Subjective and Objective Integrated)									
BC1	Measuring the compliance behavior of adolescents wearing orthopedic braces [119]	1999	Canada	40	0	10-16	Boston brace, semirigid orthopedic brace	Interview	I
BC2	A correlation study between in-brace correction, compliance to spinal orthosis and health-related quality of life of patients with Adolescent Idiopathic Scoliosis [120]	2014	China	42	0	11-15	Hong Kong brace: TLSO (underarm brace)	Chinese: TAPS, BrQ and SRS-22	I
BC3	Accuracy in the prediction and estimation of adherence to bracewear before and during treatment of adolescent idiopathic scoliosis [19]	2008	United States	108	16	10-15	Boston brace	Brace-Beliefs Questionnaire	I
BC4	The Intelligent Automated Pressure-Adjustable Orthosis for Patients With Adolescent Idiopathic Scoliosis: A Bi-Center Randomized Controlled Trial [121]	2020	China	23	0	10-14	Orthosis	SAQ, BrQ (follow up)	I
BC5	Impact of Brace-Related Stress on Brace Compliance in Adolescent Idiopathic Scoliosis: A Single-Center Comparative Study Using Objective Compliance Measurement and Brace-Related Stress [122]	2023	Japan	42	3	10-15	Underarm or Boston brace	Japanese BSSQ-brace	I
BC6	Factors Influencing Optimal Bracing Compliance in Adolescent Idiopathic Scoliosis: A Single Center Prospective Cohort Study [123]	2024	Japan	114	8	10-15	Boston brace	PROMs^ac^, SRS-22r, SJ27^ad^, JBSSQ^ae^-brace	I
BC7	Effects of Mindfulness-Based Intervention to Improve Bracing Compliance in Adolescent Idiopathic Scoliosis Patients: a Randomized Controlled Trial [124]	2023	China	62	21	10-15	Type missing (report bracing)	EQ-5D-5L, SRS-22, BrQ, ERQ-CCA^af^, SCS^ag^, GSE^ah^, PSS^ai^, FFMQ^aj^	I
BC8	Effects of Bracing in Adolescents with Idiopathic Scoliosis [5]	2013	United States	135	11	10-15.10	Rigid thoracolumbosacral orthosis	PedsQoL	I
BC9	A Randomized Controlled Trial to Evaluate the Clinical Effectiveness of 3D-Printed Orthosis in the Management of Adolescent Idiopathic Scoliosis [125]	2022	China	30	0	10.7-14.5	3D-printed orthosis	SRS-22r, TAPS, BrQ	I
RQ-A ∩ RQ-C (Subjective and Objective Integrated)									
None									
RQ-A ∩ RQ-B ∩ RQ-C (Subjective and Objective -Fully integrated with user perspective)									
ABC1	Exploration of Contributory Factors to an Unpleasant Bracing Experience of Adolescent Idiopathic Scoliosis Patients a Quantitative and Qualitative Research [126]	2022	China	13	4	10-17	Cheneau / Boston brace	Force and SRS-22, GCQ^ak^, ODI^al^	I
ABC2	Why Don’t Adolescents Wear Their Brace? A Prospective Study Investigating Psychosocial Characteristics That Predict Scoliosis Brace Wear [127]	2023	United States	37	4	10-Risser 2	Custom TLSO orthosis	Temperature, patient information from validated questionnaire	I

a RQ: research question.

b BSSQ: Bad Sobernheim Stress Questionnaire.

c SRS-22: Scoliosis Research Society-22 Patient Questionnaire.

d TLSO: Thoracolumbosacral orthoses.

e BrQ: Brace Questionnaire.

f K-BrQ: Korean version of the Brace Questionnaire.

g K-SRS-22: Korean version of the Scoliosis Research Society-22 Outcomes questionnaire.

h TAPS: Trunk Appearance Perception Scale.

i SOSORT: International Society on Scoliosis Orthopedic and Rehabilitation Treatment.

j SEQ:Self-Efficacy Questionnaire

k SAQ: Spinal Appearance Questionnaire.

l HSPQ: High School Personality Questionnaire

m 16PF: Sixteen Personality Factor Questionnaire

n BAS: Body Appreciation Scale.

o HRQoL: Health-Related Quality of Life.

p SF-36: 36-Item Short-Form Health Survey.

q CHQ PF-28: Child Health Questionnaire – Parent Form, 28 items.

r PODCI: Pediatric Outcomes Data Collection Instrument

s WRVAS: Walter Reed Visual Assessment Scale.

t POTSI: Posterior Trunk Symmetry Index.

u PedsQoL: Pediatric Quality of Life Inventory.

v RMDQ: Roland-Morris Disability Questionnaire

w BPI: Brief Pain Inventory.

x STAI: State-Trait Anxiety Inventory.

y BDI-II: Beck Depression Inventory-II.

z CAEN: corset à appui électif nocturne (“CAEN” brace)

aa EMG: electromyography.

ab VAS: Visual Analog Scale.

ac PROM: Patient-Reported Outcome Measure.

ad SJ27: Japanese Questionnaire-27

ae JBSSQ: Japanese version of the Bad Sobernheim Stress Questionnaire

af ERQ-CCA: Chinese adaptation of the Emotion Regulation Questionnaire for Children.

ag SCS: Self-Compassion Scale

ah GSE: General Self-Efficacy Scale.

ai PSS: Perceived Stress Scale

aj FFMQ: Five Facet Mindfulness Questionnaire.

ak GCQ: General Comfort Questionnaire

al ODI: Oswestry Disability Index

### Study Characteristics

#### Mapping to RQs

**Figure 2.**
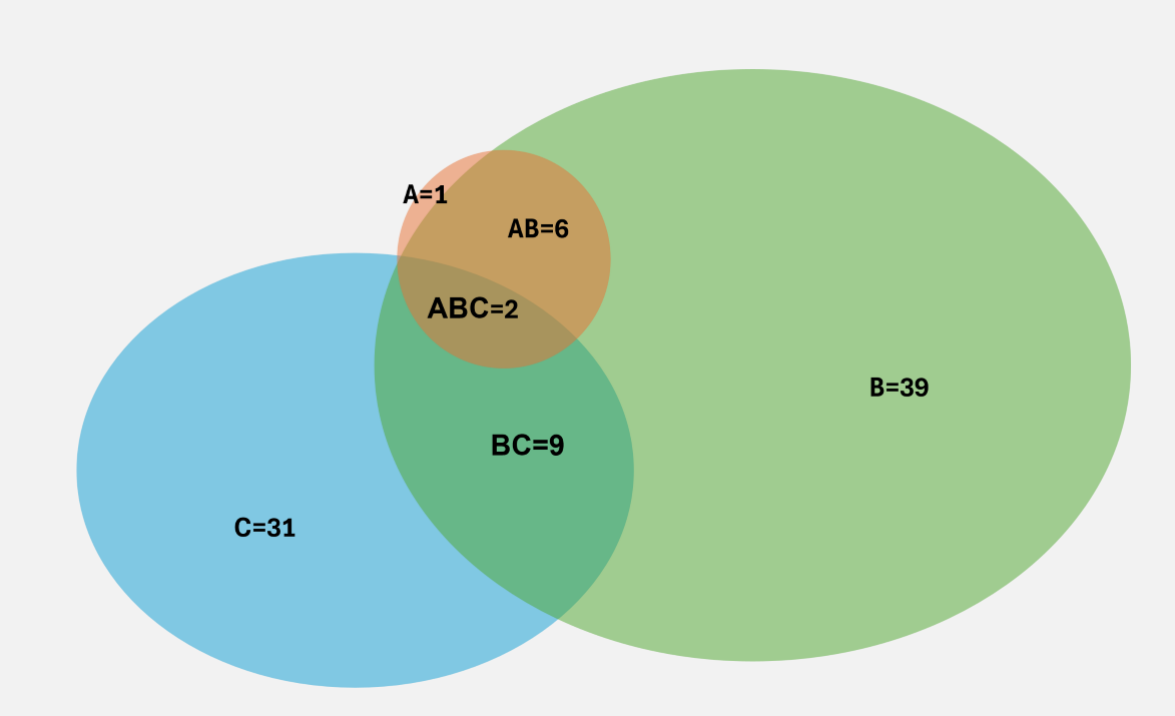
Visual illustration of study characteristics based on 3 RQs. A: User experience and perception (RQ-A), B: Monitoring Tools (RQ-B), and C: Technology-Assisted Approaches (RQ-C).

All 88 studies for data extraction were classified into 3 predefined RQs (RQs-A, B, or C; A presenting user experience and perception, B presenting monitoring tools, and C presenting technology-assisted approaches. Articles were labeled by relevancy (A, B, C, AB, BC, AC, and ABC) in the extraction table. The Venn diagram (Figure 2) illustrates the thematic overlap among the included studies. The largest group of studies (n=39, B1-B39 as listed in Column 2 of Table 1) addressed only B, focusing primarily on subjective monitoring methods such as questionnaires and self-reports, and the next largest group of 31 studies (C1-C31 as listed in Column 2 of Table 1) addressed C alone, most of which involved small-scale or pilot implementations of technologies, such as sensors and smart orthosis. Only 2 articles [126,127] addressed all 3 RQs, 6 addressed RQs A and B [113-118], and 9 addressed RQs B and C [5,19,119-122,124,125]. These distributions indicate a persistent integration gap between clinical (subjective) and technology-based (objective) monitoring in adolescent idiopathic scoliosis care. Furthermore, the results highlight that no published study meets the eligibility criteria of adolescents diagnosed with idiopathic scoliosis and aged 10-17.11 years, which integrates user experience and perception with technology-assisted approaches (RQs A and C, n=0).

Two main themes that evolved from the findings are (1) subjective (patient-reported or user experience) monitoring using questionnaires with patient-reported outcomes, interviews, and logbooks capturing the patient experience; and (2) objective (technology-assisted or sensor-based) monitoring using sensor-based systems measuring parameters such as temperature, pressure, force, and motion, and a real-time, clinically integrated support system. Integrated approaches combine both. Each included study is coded into these categories S, O, or I, as illustrated in Table 1.

#### Publication Trends

**Figure 3.**
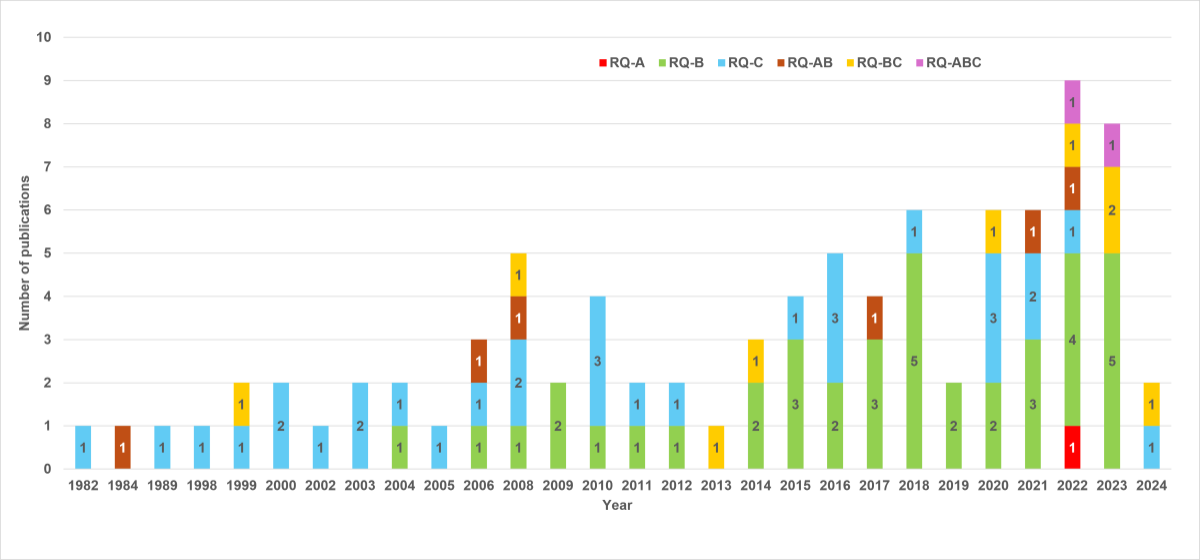
Number of publications over time based on the three research questions A, B, and C.

The number of publications in the focus area has increased over time, as illustrated in Figure 3. Technology-based monitoring has been in use for over 4 decades and accounts for 47.73% (42/88), where 42 studies are categorized into 3 subgroups (C1-C31, BC1-BC9, and ABC1-ABC2), as listed in Column 2 of Table 1. The evolution of articles over time indicates that research on technology-based monitoring has a long history, underscoring the need for updates as technology continues to advance.

Research incorporating patient-reported outcomes emerged approximately a decade later. Once these tools were adopted, an increasing number of studies began to focus on capturing previously unheard patient needs as part of the treatment burden. Articles that combined subjective monitoring tools with sensor-based approaches demonstrate a trend since 1999 toward integrating subjective (RQ A or B) and objective (RQ C) measures. Since 2022, research on user-centered integrated monitoring has gained momentum. These trends are illustrated as publications over time, with research areas and their combinations (Figure 3).

#### Global Contributors

**Figure 4.**
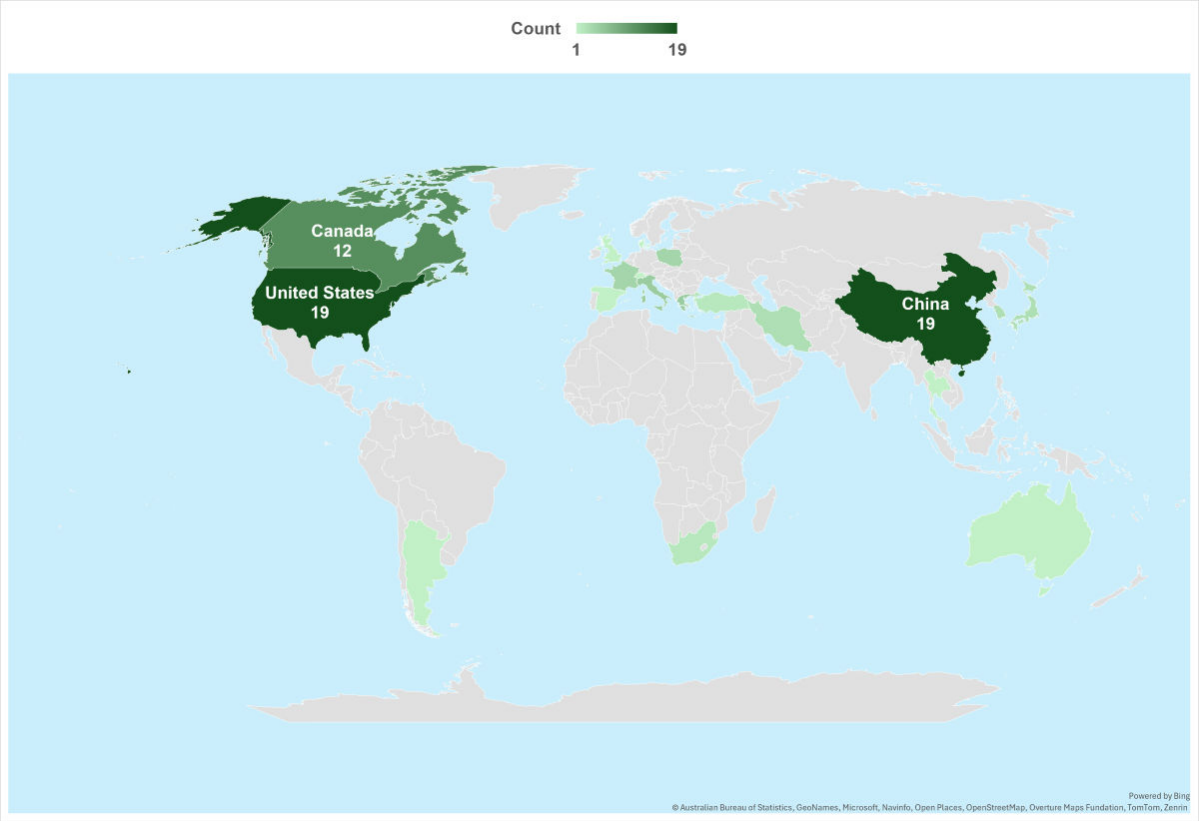
Visual representation of global contributors among the 88 included publications.

Research output was concentrated in a few countries: the United States [5,19,23,29,68,70,78,82,89,94,96,102,105,108,109,111,116,117,127] and China [30,46,47,58,62,75-77,84,88,92,93,100,115,120,121,124-126] (n=19 each) contributed the most, followed by Canada [57,63,86,87,91,95,98,99,103,107,110,119] (n=12); other contributors included Italy [61,71,72,81,97] and Greece [49,60,67,85,114] (n=5 each), France [90,104,112,118] and Poland [51,69,74,79] (n=4 each), Iran [48,50,65], Japan [113,122,123], and South Korea [54,55,56] (n=3 each), South Africa [59,64], and Turkey [53,80] (n=2 each), and Argentina [66], Australia [83], Denmark [73], Spain [52], Switzerland [101], Thailand [22], and the United Kingdom [106] (n=1 each; Figure 4 and Table 1).

The observed trends indicate that research in multidisciplinary health and technology domains with a user-centered focus is concentrated in a few leading countries, such as China, the United States, and Canada, with small contributions from other regions. Subjective and objective monitoring with the patient as a participant requires approval in accordance with the relevant country’s ethical guidelines. Geographic distribution shows the ethical constraints and regulatory challenges in conducting studies that integrate health and technology among participants diagnosed with diseases, such as idiopathic scoliosis.

#### Orthosis Types

Across the 88 studies, a range of rigid and soft orthoses was reported, including Boston [60,89,103,107,116,119], Milwaukee [65,88], Chêneau [30,71,74,104], and Rigo System Chêneau [59,64], along with various Thoracolumbosacral orthoses, such as rigid [70,72], underarm [120], and other types include the Sforzesco brace [71], Providence [73,102], Charleston, Wilmington [23,96], SpineCor [115], dynamic derotation brace [85], CAEN brace [90] and asymmetric Scoliosis Brace by SLC clinic [49] as well as fiberglass [97], custom-fabricated [76], 3D-printed [125], garment [100], and semirigid [119]. Unfortunately, some articles [114,118] report on the use of braces or orthosis without specifying the type. Some articles report the use of orthosis without reporting the exact type or other specifications (eg, studies by Lin et al [121,125]), while some full-text reports the patient’s use of orthosis. For example, the study by Sapountzi-Krepia et al [114] mentions that the orthosis was worn for more than 12 hours, while Bertuccelli et al [81] report on orthotic treatment. Details from all included articles are reported in Table 1. The orthosis used in that study will influence participants’ perspectives and patient-reported outcomes. Given the broad scoping focus on analyzing generic challenges in scoliosis orthotic management, we consolidated the observations based on the evolved themes.

#### Sex

**Figure 5.**
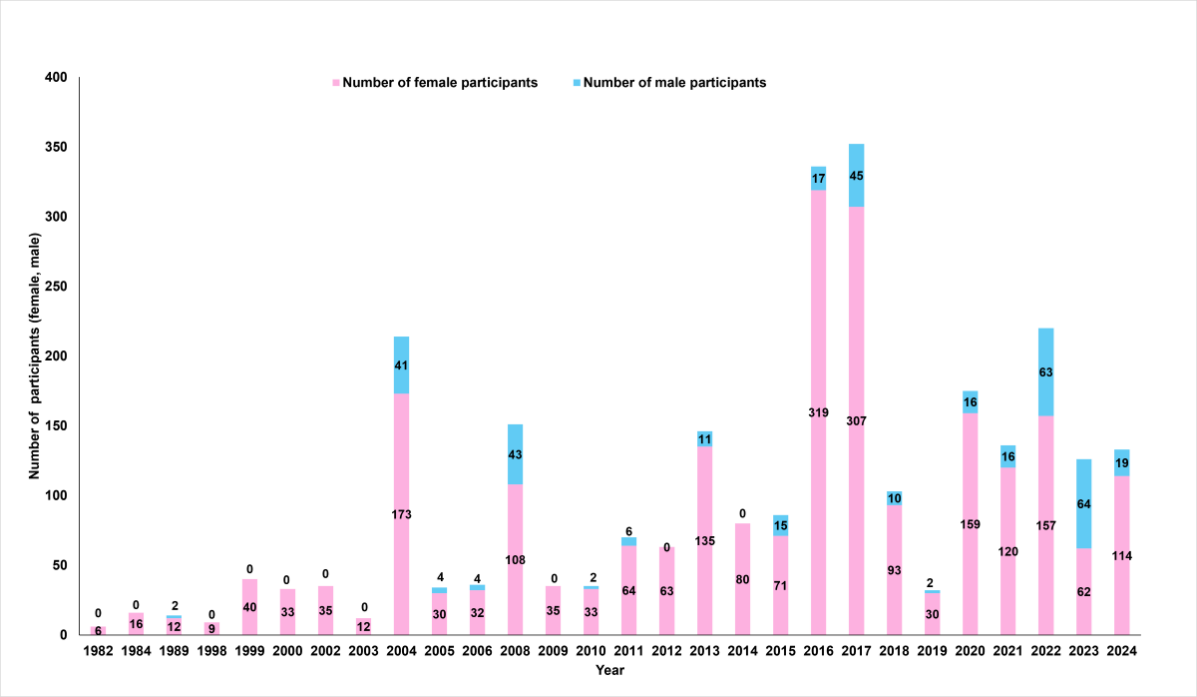
Sex distribution trend of all included articles.

Sex distributions broadly reflect the higher female prevalence [1,2], with negligible male representation until 2004 and increases thereafter (Figure 5). Although male individuals remain underrepresented overall, their contribution to research has improved since 2004. Studies that explore sex-specific needs and perspectives have yet to be reported in detail. The use of patient-reported tools in research has stimulated researchers since 2004 to capture the distinctive needs of male individuals, which differ from those of female individuals, especially during adolescence. In 1 study that used a comparison of bracing versus observation reported no significant overall health-related quality of life (HRQoL) reduction with bracing; however, they reported about family-activity difference favoring male individuals [78]. More focus is needed to know sex-based monitoring preferences in the future, especially for adolescents treated with orthoses.

### Subjective Monitoring (RQ A and RQ B)

Subjective monitoring in the management of adolescent patients with idiopathic scoliosis primarily evolved from research on patients’ lived experience and perceptions, as reflected in RQ A and traditional clinical monitoring approaches relevant to RQ B. These strategies use different tools to capture patients’ needs. For example, clinicians rely on self-reports, relevant questionnaires to address needs (eg, stress or quality of life), and interviews to capture user perceptions. Over time, they have become central to understanding psychosocial and cultural factors influencing orthosis compliance.

#### Questionnaires as a Subjective Monitoring Tool

Questionnaires and patient logbooks capture user compliance and user-reported data that support subjective scoliosis management for adolescent patients with idiopathic scoliosis [76]. Studies verify that exercise with bracing is associated with improvements in quality of life and functional outcomes, using PROMs or logbooks [58,62,63]. Comparative studies recorded efficacy differences between rigid versus flexible orthoses and captured discomfort and acceptance via a visual analog scale questionnaire, along with open-ended items [128]. The use of “open-ended” items in questionnaires, along with other sensor monitoring in a mixed design, was found beneficial for evaluating patient needs [124]. A combination of subjective data from interviews with sensor-based objective data reports about mal-appearance, inconvenience, and psychological distress [126]. Future studies should integrate PROMs with objective sensor-derived data to provide a more comprehensive understanding of user experiences and treatment adherence.

Among the included studies, subjective monitoring reports the use of Scoliosis Research Society-22 Patient Questionnaire, Brace Questionnaire, Bad Sobernheim Stress Questionnaire (Brace/Deformity), Italian Spine Youth Quality of Life, EQ-5D-5L, and 36-Item Short-Form Health Survey [51,52,55,67,71,75,76,124]. A multicenter study found that adolescents with more severe scoliosis underestimated their deformity and body image. Perception tools Walter Reed Visual Assessment Scale, Spinal Appearance Questionnaire, and Trunk Appearance Perception Scale were also used as a clinical tool to capture user perception [118]. The medium of communication to express influences subjective monitoring. The language of the questionnaire tool, including verbal and pictorial illustrations, is used to capture the user’s perspective on the disease condition, and cultural adaptation of questionnaires based on the research focus can influence the expression of that perspective. Other main instruments included Pediatric Quality of Life Inventory [82], Posterior Trunk Symmetry Index [80], Brief Pain Inventory [83], and psychological scales (State-Trait Anxiety Inventory, Beck Depression Inventory-II) [84] to capture often unexpressed psychological aspects of adolescent patients with idiopathic scoliosis burden.

#### Cross-Cultural Adaptations of Monitoring Tool

Cross-cultural adaptation of the standardized questionnaires is the strategy used to improve user acceptability and relevance among culturally diverse populations. Among the articles included in the review, 6 studies adapted the Bad Sobernheim Stress Questionnaire from the base language to another target language (Japanese, Spanish, Persian, Greek, Polish, and Chinese) [47-49,51,52,113], 2 adapted the Brace Questionnaire (Turkish and Korean) [53,56], and 1 each adapted to Korean Quality of Life Profile for Spine Deformities [54], Korean Italian Spine Youth Quality of Life [55]. Across these studies, the Scoliosis Research Society Patient Questionnaire-22 (and its revised version) was commonly used as a reference instrument for comparative analysis. The adaptation processes follow an established methodological process, incorporating forward-backward translation, expert review, pilot testing, and evaluation that includes test-retest reliability, internal consistency, and validity with reference tailored to the study objectives (eg, study by Jafarian et al [48]). Collectively, these articles address RQ B by demonstrating the utility of culturally adapted questionnaires for monitoring, and RQ A by capturing patient perceptions within routine care.

#### User Perceptions

Given the scoping focus on analyzing long-term challenges in scoliosis orthotic management, we consolidate generic user experience and perceptions. In a qualitative study, patient interviews revealed that they were afraid of what the doctors might say during the next feedback session [114]. Orthosis compliance and outcomes vary based on the orthosis used by the participant. For instance, the Milwaukee brace, a type of orthosis, was reported to cause emotional strain and barriers, including issues with clothing fit, restricted movement, and social limitations [117]. Across the included studies, physical and emotional challenges related to orthosis wear were consistently noted: discomfort, restricted movement, heat or climate-related issues, clothing difficulties, and social limitations. The only article from RQ A that used patient-reported data from in-depth semistructured interviews with Chinese adolescents who described activity- and heat-related discomfort and a desire for more personalized, co-designed orthoses. Active patient participation in the intervention is a recommendation [46]. Chinese randomized trial reports the use of a specially designed thin undergarment that improved comfort and compliance [76]. Although the study was conducted in China, similar climatic and seasonal conditions may also influence orthosis use in patients from other geographical regions. Family dynamics (eg, parental reminders) also influenced adherence and contributed positively to scoliosis management among adolescents. These patterns address RQ A user experience and perceived barriers or facilitators, and RQ B, especially the use of clinical tools such as logbooks to monitor adherence.

#### Psychological or Emotional Well-Being

Bracing in adolescent patients with idiopathic scoliosis has a significant impact on HRQoL, reporting psychological, motor, social, and school-related challenges [66]. Studies use tools, for example, Body Appreciation Scale (BAS) and trunk appearance perception scale to examine body aesthetics and user perception that considers self-image, trunk perception, and psychological outcomes [68,118]. Reduced body-related attentional biases are reported with the use of psychotherapy [81]. Long-term orthotic treatment is demanding. Still, progress in trunk correction and self-visualization offers a sense of positivity. Studies have demonstrated an optimistic view of bracing when studied using the trunk appearance perception scale as orthoses can influence trunk correction, leading to greater satisfaction with orthotic treatment [61]. Likewise, using another visualization score, the BAS gave a similar perspective from the orthosis-treated and control group [68]. This underscores the need for interdisciplinary psychological and social support for adolescent patients with idiopathic scoliosis.

#### Family Context

The patient’s family also played a significant role in handling challenges and managing scoliosis, as reported in the articles included. In Argentina, a study using a brace questionnaire recommended separate patient and parent surveys to reduce response bias [66]. Polish studies using Bad Sobernheim Stress Questionnaire-brace or deformity found comparable stress in adolescents and parents, with parents overestimating deformity-related stress; spinal appearance questionnaire comparisons emphasized the roles of both mothers and fathers [69,74]. Accepting cultural diversity, the roles of both parents, mother and father, and family bonding are recognized as influential parameters for adolescents’ emotional coping.

#### Societal or Socioeconomic Context

Subjective tools identified peer perceptions at school as sources of stress or anxiety [113]. In a Spanish cohort using Bad Sobernheim Stress Questionnaire-brace, no embarrassment was reported among adolescents wearing Rigo System Chêneau orthosis with close family and friends at school [52]. Financial burdens were noted (orthosis and consultation costs) [51]. Rural versus urban comparisons showed similar moderate orthosis-related stress and low deformity stress, with greater appearance concern among rural participants with larger lateral shift [79].

To sum up the findings, addressing RQs A and B by mapping psychosocial parameters relevant to subjective monitoring (eg, sex, well-being, family, and school or society) influences patient perspectives on stress, body image, and daily life.

### Objective Monitoring (RQ-C)

#### Sensor Types and Parameters

Early systems (1980s) were developed in controlled settings with static assessments. Over time, objective monitoring expanded to include electromyography (EMG), pressure or force, temperature, and motion sensors that captured orthosis use, activity, and physiological responses.

#### EMG

EMG is used to evaluate neuromuscular responses to bracing. There are limited (n=3) studies using EMG. The oldest included study, from 1982 in Canada, reported that it was unable to detect any observable trend among the 6 female individuals [110]. Even though the results were not promising, objective monitoring to understand the effect of orthosis began to evolve. Moreover, 2 other studies that used EMG demonstrated how orthosis improved spinal stability by stimulating muscle engagement [95] and the feasibility of wearable EMG sensor-based posture tracking for daily activities [111].

#### Motion

Movement tracking represents activity in daily life; 1 study reported no significant difference in walking activity between adolescent patients with idiopathic scoliosis and controls using a smartphone-based approach [112]. Multimodal systems combining accelerometers, gyroscopes, and force are gaining research interest recently [29].

#### Pressure or Force

Studies monitored strap or pad forces and interface pressures to support fit, personalization, and compliance, ranging from calibrated measurements to embedded or load-monitoring systems and mapping approaches linking tension, pressure, and correction [103,106,107,129]. One system reported a decline in tightness without monitoring and improved compliance with monitoring or refitting [29]. Works characterized different research interests that include corrective force patterns [87,89], explored respiratory mechanics under bracing [93], examined posture-specific force changes [85], and used exercise-related pressure patterns to inform personalized exercise selection [97]. Adjustable orthoses logged pressure and temperature over extended periods [121].

#### Temperature

Temperature sensors quantified wear time with high accuracy and were used to explore adherence patterns. Prospective cohorts recorded orthosis wear at set intervals over months and reported associations between higher compliance and improved outcomes, with features for time-based feedback, as reported in [23,96,105]. Other studies that use temperature-based reporting monitoring report high accuracy in estimating daily wear, increased daily wear with compliance counseling, and associations between reduced adherence and curve progression in night-time bracing [102,108,109]. Studies compared orthosis types, estimated compliance in dynamic systems, and examined temporal patterns (for eg, higher weekday or night-time wear; relationships with stress and satisfaction) [5,19,101,122,123,125]. Together, these sensor families demonstrate the feasibility of objective monitoring of muscle activity, strap or pad forces and interface pressure, wear time, and movement or activity during orthotic treatment.

#### Wear Time and Adherence

In temperature-based studies, wear time was derived from periodic logging and compared with diaries and/or clinical outcomes; high accuracy versus diaries was reported, and adherence counseling and visit-time feedback were associated with increased wear [23,96,108,109]. Findings describe differences by time period (eg, weekday or night-time patterns), declines in orthosis tightness over time without monitoring, and improved adherence with feedback-enabled systems [29,122]. In night-time bracing, lower adherence was associated with curve progression [102]. Large cohort data supported a dose-response relationship between longer wear and reduced progression to surgical thresholds [5]. These studies address RQ C by quantifying orthosis wear time and adherence patterns, and by relating adherence to treatment outcomes.

#### Integration of Subjective and Objective Monitoring

Integrated clinical monitoring, along with relevant data visualization, is gaining importance due to its design strength. Integrated monitoring of adolescents with idiopathic scoliosis orthotic treatment combines subjective patient-reported outcomes with objective sensors to track both perceived burden and real-time adherence. Integrated monitoring combines 9 articles with RQs B and C, which include compliance monitoring and interviews, as well as a randomized controlled trial using automated pressure-adjustable orthoses and variant temperature or heat sensors, linking wear quality with HRQoL outcomes [5,19,119-125]. Moreover, 2 studies that demonstrate the integration of 3 RQs with an all-around monitoring approach that merges user perspectives with clinical and technological approaches, namely qualitative interviews and psychosocial questionnaires, to capture lived experience [126,127]. Integration of subjective and objective monitoring is progressing.

#### Real-Time Monitoring

This scoping review tries to collect evidence from all selected databases up to the search date, which allowed the inclusion of engineering articles from the 1980s and 1990s. These early studies reflect efforts to measure orthosis adherence objectively; however, they relied on nonportable devices, limiting monitoring to controlled laboratory environments. Research growth is an indicator of how technology and health research are advancing over time, with a growing focus on user-focused solutions that include portable and compact solutions. At the same time, objective sensors and adherence logs provide real-time data. Integration of patient needs, cultural context, and comfort, along with biomechanical and compliance metrics, can provide assurance in scoliosis management and serve as a convergence that enables accurate adherence measurement, personalized feedback, and improved clinical decision-making for patient-centered care.

One configuration involved force sensors with a mobile mini-program for daily uploads, enabling remote clinician monitoring and scheduled follow-ups, and was evaluated in 28 adolescents, with satisfaction assessed at 6 months [30]. The design load-monitoring system for objective monitoring developed by Lou et al [98] tracks both the quantity and quality of orthosis wear. The combination of objective data supported with PROMs both supports adherence and patient-reported experience [124]. These findings demonstrate the feasibility of real-time or connected monitoring with clinic-linked follow-up, complementing subjective tools to provide a more complete view of orthosis use.

These innovations target continuous data collection and personalized solutions that help health personnel and empower adolescent users to play an active role in their treatment, reducing the effort required of health personnel [26,30,31]. Advancements could lead to patient-friendly, effective scoliosis management and improve long-term outcomes with more focus on distinct aspects of monitoring adolescent patients with idiopathic scoliosis.

## Discussion

### Summary of Key Findings by RQs and Themes

The review identified 2 central themes, namely subjective monitoring and objective monitoring, with limited integration. RQs A and B capture subjective monitoring that relies on patient-reported outcomes and qualitative user-experience data, consistently documenting discomfort, restricted movement, heat or climate concerns, clothing challenges, peer-related anxiety, and the influence of family dynamics. Cross-cultural adaptations were common and used standard translation procedures. The exercise program in combination with orthotic treatment improves quality of life as well as body function. The RQ C, objective monitoring demonstrated feasibility using temperature (wear-time quantification), pressure or force (fit, personalization, and corrective load), EMG-based neuromuscular response, and motion (activity or posture). Temperature-based monitoring showed high accuracy for wear-time estimation and was associated with increased adherence when coupled with counseling or feedback; dose-response patterns linked greater wear with reduced progression. The strict age restrictions, along with the exclusion of studies involving mixed-age idiopathic scoliosis cohorts, probably narrowed the evidence base and reduced the inclusion of potentially relevant sensor-based research. Connected systems enabling remote follow-up were feasible in small samples.

Clearly distinguishing between subjective and objective monitoring approaches is clinically meaningful. While objective tools offer precise and continuous measurements of orthosis wear and biomechanical parameters, they do not capture how adolescents experience orthotic treatment in daily life. Conversely, patient-reported measures provide critical insight into comfort, usability, and psychosocial impact but may be influenced by recall bias or social desirability. Integrated approaches that combine both perspectives appear particularly promising, as they acknowledge that treatment adherence and effectiveness are shaped by both measurable behaviors and lived experience. This framework helps contextualize current evidence and highlights directions for future digital health development in scoliosis care.

### Interpretation and Clinical Implications

Different PROMs and qualitative tools capture adherence to comfort, climate, clothing, and psychosocial context, which are not accessible from imaging alone, while sensor-based systems provide objective quantitative data on wear time and the quality of orthosis wear. A combination of diverse modalities supports individualized adjustments (eg, orthosis fit or tightness and targeted exercises) by providing tailored psychosocial support. Family involvement and feedback mechanisms were associated with better adherence.

Mixed methods designs that link temperature-based adherence with HRQoL instruments illustrate how clinicians can monitor both use and impact to guide timely interventions. Although Thermobrace was excluded (due to participants with a diagnosis of hyperkyphosis along with remaining idiopathic scoliosis), temperature-based approaches, in general, were used and reported accurate wear-time estimation [27]. A recent Chinese pilot study reports the use of an integrated sensor, namely force and temperature, that provides a comprehensive understanding of the compliance by demonstrating data from 12 patients by comparing subjective and objective monitoring for 1 month and found that patients’ self-reported data exceeded the sensor’s actual average wear time [24]. User acceptance of objective monitoring provides reassurance and perceived helpfulness as a long-term supportive tool [17]. A recent study reports that subjective patient-reported data, along with automated alerts to clinicians at predefined thresholds, can increase usability and engagement, supporting users in meeting prescribed orthosis-wear recommendations during the study period [130].

### Implications for Research and Future Directions

Evidence collected over time across multidisciplinary technological and clinical research suggests a gap for integrated user-centered approaches. Studies need to include standardized monitoring protocols, detailed reporting of clinically relevant participant demographics, and harmonized outcomes, which would improve comparability and inclusiveness of research across disciplines. In the future, subjective monitoring questionnaires are to be culturally adapted and validated, especially focusing on orthotic treatment (eg, study by Caronni et al [131]). Research could also expand sex-specific analyses, evaluate acceptability across cultures, and test multimodal systems (eg, temperature, pressure, or motion) in larger, longitudinal cohorts that support patient rehabilitation.

An integrated workflow can combine subjective PROMs with objective technology-based monitoring, supported by structured clinical interventions and timely feedback, to enhance adherence throughout treatment and patient rehabilitation [130]. For example, real-time review could empower adolescent users and their families by enabling earlier identification of nonadherence and supporting personalized treatment adjustments. The system needs to ensure user and patient security and trustworthiness, and provide patient-specific guidance [132], in line with the nation’s protocols.

The continued gap in integrating subjective patient experiences with technology-assisted monitoring might be due to structural barriers, such as the stages of technology readiness of emerging innovations for integration into clinical workflows, existing ethical complexities in clinical trial approval processes, and user acceptance and trust in new technologies. The integration of subjective and objective monitoring advances slowly. Technological evidence must be rigorously validated before it can be used in a clinical trial. Thus, innovative technologies are typically evaluated through phased testing to improve technology readiness. Objective sensor data (eg, body-pressure distribution on a scoliosis orthosis) are tested on a mannequin or a laboratory-based test environment with clinically relevant pressure patterns, serving as the preparatory analysis for the future clinical trial [133]. These stages of testing and preparation ensure feasibility, safety, and effectiveness.

Standardized protocols should be established for data handling and clinically aligned technology-based monitoring to support personalized, data-driven care for adolescents with idiopathic scoliosis. Detailed testing has been conducted and reported to prepare for future clinical testing of a real-time contact pressure monitoring solution and highlights that future monitoring solutions for adolescent idiopathic scoliosis during bracing need to consider patient comfort [134].

Future research directions across the 3 RQs highlight critical gaps and opportunities. For RQ A, there is a need to strengthen the focus on lived experiences and user perceptions, which remain the least represented in the current literature. Currently, no existing review integrates (RQ A) user perspectives, wishes, and needs with technology-based monitoring (RQ C) in the context of adolescent idiopathic scoliosis. For RQ B, monitoring tools should evolve to include questionnaires that address technological perspectives, usability challenges in daily life, concerns related to personal and health data security, and new needs. For RQ C, technology updates should be integrated into the health care system that uses real-time monitoring and time-bound clinically significant feedback. In the future, co-design of systems with stakeholders can help to develop a user-friendly solution that can be widely accepted and suitable for rigorous use.

### Challenges

Methodological diversity among the included articles complicated appraisal using established tools Critical Appraisal Skills Program, Joanna Briggs Institute, and Mixed Methods Appraisal Tool, which were not compatible with engineering-focused studies that often lack a clinical structure, underscoring the need for frameworks that bridge engineering and clinical research. Variability was observed in the reporting of CHERRIES (Checklist for Reporting Results of Internet E-Surveys) checklist items related to methodological details across included studies, with more comprehensive reporting in health science–focused research (eg, cultural adaptation of questionnaires) and comparatively limited descriptions in technology-focused studies, where standardized questionnaires were often used as data collection tools secondary to technological evaluation [135]. Informal appraisal without formal methodological appraisal proceeded alongside iterative data coding, which is acceptable in scoping reviews.

Inconsistent reporting of participant details (age, diagnosis, and orthosis type) was more common in engineering papers; lack of focus on RQs and emphasis on technology alone led to exclusions. Along with orthosis monitoring, soft orthosis, including garment orthosis and vests, were included in the scope. However, due to the strict requirements for patient-based studies, many studies were excluded because they often lacked patient participation criteria. Only patient-tested solutions were retained, which led to the exclusion of several early-stage prototypes under RQ C.

### Strengths

Having a specific and focused target group, adolescents diagnosed with idiopathic scoliosis, led to the exclusion of technologically noteworthy studies. On the other hand, this focus strengthened the scoping review’s ability to identify gaps and highlight future needs toward interdisciplinary research. The inclusion of diverse article types spanning medical and engineering fields enriched the analysis. The 2 independent reviewers, one with a medical background and the other with a technological background, helped with this process. Screening benefited from Rayyan-supported comparisons; however, final inclusion and exclusion decisions were made manually, using human-judgment-guided selection.

### Limitations

This review is limited by sample size constraints, lack of longitudinal data, poor reporting in engineering studies, and insufficient risk of bias evaluation, which may affect the robustness of the evidence base. The review included articles from the listed databases that are digitally available and English-language publications, leading to the exclusion of relevant studies that were published in other language publications, nondigital form, or sourced from unindexed repositories.

Our strict age inclusion criteria (10‐17.11 y) excluded multiple technology-focused papers with mixed-age samples, likely reducing the number of eligible sensor-based studies. Clinically significant parameter Cobb angle was considered during protocol development. However, a broader approach was adopted to accommodate variability in reporting, especially in nonhealth publications.

### Conclusions

This scoping review of 88 included studies reveals the clear growth in adolescent patients with idiopathic scoliosis conservative monitoring from separate subjective and objective approaches toward integrated, patient-centered models. Questionnaire-based tools remain essential for capturing psychosocial burden, body image, and cultural context, but they cannot measure real-time adherence or biomechanical quality. Technological solutions that use sensors provide objective information, progressing from static laboratory setups to portable, connected systems that enable remote oversight and timely feedback. Subjective tools and objective sensors provide complementary views of adolescent orthosis wear. Combining patient-reported outcomes with sensor data offers the greatest potential, as it provides a multidimensional view of adherence, comfort, and clinical effectiveness. However, reporting inconsistencies and limited scalability persist, especially in technology-focused research. In the future, research should better integrate health and technology disciplines, integrate culturally adapted objective and subjective metrics, and co-design a user-centered solution that motivates adolescents to ensure usability. Moving beyond pilot studies to mixed method clinical trials and real-time platforms will enable personalized care, improve adherence, and optimize outcomes. The convergence of subjective and objective monitoring marks the shift toward holistic management of adolescent patients with idiopathic scoliosis, aligning clinical precision with patient motivation.

## Supplementary material

10.2196/88880 Multimedia Appendix 1 Literature search documentation.

10.2196/88880 Multimedia Appendix 2 Data items.

10.2196/88880 Checklist 1 PRISMA ScR checklist.

## Abbreviations

BAS: Body Appreciation Scale
CHERRIES: Checklist for Reporting Results of Internet E-Surveys
CONSORT-EHEALTH: Consolidated Standards of Reporting Trials of Electronic and Mobile Health Applications and Online Telehealth
EMG: electromyography
HRQoL: health-related quality of life
PICOS: Population, Intervention, Comparison, Outcome, and Study design
PRISMA-ScR: Preferred Reporting Items for Systematic Reviews and Meta-analyses Extension for Scoping Reviews
PROM: patient-reported outcome measure
RQ: research question
SOSORT: International Society on Scoliosis Orthopedic and Rehabilitation Treatment
