# Supplementary material for: Patient-Reported and Technology-Assisted Monitoring in Orthotic Management of Adolescents With Idiopathic Scoliosis: Scoping Review
Source: JMIR Mhealth Uhealth. 2026 Aug 10;14:e88880. doi: 10.2196/88880 (PMC13455584; doi:10.2196/88880)
Supplement: Supplementary file 1 [file mhealth-v14-e88880-s001.docx]

UNIVERSITETSBIBLIOTEKET, RESSURSGRUPPE FOR LITTERATURSØK

**Literature search documentation**

**Project:**

The scoping review aims to identify and map the available evidence for the methodology and application of intelligent vests for scoliosis brace treatment of AIS.

**Literature search conducted by:**

Ingjerd Legreid Ødemark & Elisabeth Karlsen

**Search requested by:**

Anna Mathew, Kamila Sykorova, Parisa Gazerani,

Nenad Pavel, Marianne Bakke Johnsen,

Minna Pikkarainen Sadeeq Ali, Mette Fløystad Kvammen.

**Date:**

23.05.2024

# **Summary of the search information**

**Results**

| *Databases* | *Results* |
| --- | --- |
| *Medline via Ovid* | *485 + 531* |
| *Embase via Ovid* | *681 + 791* |
| *PsycInfo via Ovid* | *8 + 22* |
| *Cochrane Library via Wiley* | *71 + 107* |
| *Cinahl vi EBSCOhost* | *238 + 280* |
| *Web of Science Core Collection* | *348 + 450* |
| *Scopus* | *640 + 818* |
| *Total including duplicates* | *5470* |

# **Development of the literature search strategy**

The search strategy is two-part due to the challenge of having several research questions that need to be answered.

(1) The main research question is about various aspects related to ***smart vests* and *scoliosis***. These two elements are constructed with alternative search words (subject headings/text words) that are combined with OR. Next, the different aspects are combined with AND. This means that one or more words within each aspect must appear in either the title, abstract, or subject headings of a reference for the article to be included in the result list. No further aspects have been added to the search due to manageable search results. This will cover articles about all aspects of *smart vests* and *scoliosis*, such as user experiences, if the article contains the search terms on these two aspects included in the strategy.

(2) However, the client also wants research on *user experience* regarding just the *brace*, without the need for a smart aspect to be present. For this reason, an additional search on the aspect ***brace* and *user experience*** has been conducted. This means that there are two strategies in each database, which will (together with searching several databases) cause an overlap with multiple similar references (duplicates) in the result lists. These duplicates will be removed using the reference tool.

# **Search history**

1. Search history 1: scoliosis AND smart vests
2. Search history 2: scoliosis AND braces AND user experiences

Search history 1: scoliosis AND smart vests

**Database**:	Ovid MEDLINE(R) Epub Ahead of Print and In-Process, In-Data-Review & Other Non-Indexed Citations and Daily

**Date**:	21.05.2024

**Limits:**	-

**Results**:	485

| **#** | **Searches** | **Results** |
| --- | --- | --- |
| 1 | Scoliosis/ | 21453 |
| 2 | scolios*.tw,kw,kf. | 26760 |
| 3 | 1 or 2 | 30873 |
| 4 | orthotic devices/ or braces/ | 12734 |
| 5 | (brac* or corset* or orthos* or garment* or undergarment* or velcro or band* or belt* or fabric* or textile* or vest or vests or t shirt* or suit* or wearable* or cloth* or patch or stretchable*).tw,kw,kf. | 1436582 |
| 6 | 4 or 5 | 1442047 |
| 7 | Mobile Applications/ or microcomputers/ or computers, handheld/ or smartphone/ or minicomputers/ | 38383 |
| 8 | Telemedicine/ | 39908 |
| 9 | (sensor or sensors or electronic* monitoring or digital* monitoring or scolios* track* or mulitplexed sensing or microfluidic device*).tw,kw,kf. | 233258 |
| 10 | (scolios* adj3 application*).tw,kw,kf. | 41 |
| 11 | (mobile application* or app or apps or mapp or eapp or microcomputer* or micro computer* or minicomputer* or mini computer* or smartphone* or smart phone* or handheld device*).tw,kw,kf. | 81150 |
| 12 | (mhealth or m health or mobile health or ehealth or e health or electronic* health or telemedicine or tele medicine or telehealth or tele health).tw,kw,kf. | 94818 |
| 13 | human robot* interfac*.tw,kw,kf. | 88 |
| 14 | ((soft or dynamic* or real time or remote or continous* or activity) adj2 (sensor* or sensing or device* or electronic* or technolog* or computer* or instrument* or system* or monitor* or robot* or track*)).tw,kw,kf. | 131968 |
| 15 | or/7-14 | 530561 |
| 16 | 6 and 15 | 80052 |
| 17 | Wearable Electronic Devices/ | 8857 |
| 18 | (e textile* or etextile* or electronic skin* or eskin* or e skin*).tw,kw,kf. | 1706 |
| 19 | ((intelligen* or electro* or smart or flexib* or digital* or sensor* or sensing or technolog* or monitor* or software* or soft ware* or robot* or biosensor* or biosignal* or biomarker* or biometrics* or biomechanic* or active or soft*) adj5 (brac* or corset* or orthos* or garment* or undergarment* or velcro or band* or belt* or fabric* or textile* or vest or vests or t shirt* or suit* or wearable* or cloth* or patch or material* or stretchable*)).tw,kw,kf. | 207085 |
| 20 | ((wearable* or body* or posture) adj2 (sensor* or sensing or device* or electronic* or technolog* or computer* or instrument* or system* or monitor* or robot* or track*)).tw,kw,kf. | 39103 |
| 21 | or/16-20 | 270530 |
| 22 | 3 and 21 | 485 |

**Database**:	Embase <1974 to 2024 Week 20> via Ovid

**Date**:	21.05.2024

**Limits:**	-

**Results**:	681

| **#** | **Searches** | **Results** |
| --- | --- | --- |
| 1 | idiopathic scoliosis/ or adolescent idiopathic scoliosis/ or scoliosis/ | 39876 |
| 2 | scolios*.tw,kw,kf. | 35056 |
| 3 | 1 or 2 | 44776 |
| 4 | spinal brace/ or boston brace/ or jewett brace/ or milwaukee brace/ or pelvic belt/ or thoracolumbosacral orthosis/ | 1257 |
| 5 | corset/ or lumbosacral corset/ | 700 |
| 6 | brace/ or posture correction brace/ | 10303 |
| 7 | orthosis/ | 8276 |
| 8 | (brac* or corset* or orthos* or garment* or undergarment* or velcro or band* or belt* or fabric* or textile* or vest or vests or t shirt* or suit* or wearable* or cloth* or patch or stretchable*).tw,kw,kf. | 1663876 |
| 9 | 4 or 5 or 6 or 7 or 8 | 1670997 |
| 10 | wearable computer/ or wearable device/ or microcomputer/ or personal digital assistant/ or smartphone/ or minicomputer/ | 50842 |
| 11 | wearable sensor/ | 3295 |
| 12 | telemedicine/ or telehealth/ or telemonitoring/ | 71186 |
| 13 | mobile application/ or mobile health application/ | 28378 |
| 14 | (sensor or sensors or electronic* monitoring or digital* monitoring or scolios* track* or mulitplexed sensing or microfluidic device*).tw,kw,kf. | 248903 |
| 15 | (scolios* adj3 application*).tw,kw,kf. | 61 |
| 16 | (mobile application* or app or apps or mapp or eapp or microcomputer* or micro computer* or minicomputer* or mini computer* or smartphone* or smart phone* or handheld device*).tw,kw,kf. | 106250 |
| 17 | (mhealth or m health or mobile health or ehealth or e health or electronic* health or telemedicine or tele medicine or telehealth or tele health).tw,kw,kf. | 123989 |
| 18 | human robot* interfac*.tw,kw,kf. | 76 |
| 19 | ((soft or dynamic* or real time or remote or continous* or activity) adj2 (sensor* or sensing or device* or electronic* or technolog* or computer* or instrument* or system* or monitor* or robot* or track*)).tw,kw,kf. | 156552 |
| 20 | or/10-19 | 637236 |
| 21 | 9 and 20 | 83773 |
| 22 | wearable device/ or wearable sensor/ | 5472 |
| 23 | (e textile* or etextile* or electronic skin* or eskin* or e skin*).tw,kw,kf. | 1457 |
| 24 | ((intelligen* or electro* or smart or flexib* or digital* or sensor* or sensing or technolog* or monitor* or software* or soft ware* or robot* or biosensor* or biosignal* or biomarker* or biometrics* or biomechanic* or active or soft*) adj5 (brac* or corset* or orthos* or garment* or undergarment* or velcro or band* or belt* or fabric* or textile* or vest or vests or t shirt* or suit* or wearable* or cloth* or patch or material* or stretchable*)).tw,kw,kf. | 213604 |
| 25 | ((wearable* or body* or posture) adj2 (sensor* or sensing or device* or electronic* or technolog* or computer* or instrument* or system* or monitor* or robot* or track*)).tw,kw,kf. | 45094 |
| 26 | or/21-25 | 284150 |
| 27 | 3 and 26 | 681 |
|  | | |

**Database**:	APA PsycInfo <1806 to May Week 3 2024>via Ovid

**Date**:	21.05.2024

**Limits:**	-

**Results**:	8

| **#** | **Searches** | **Results** |
| --- | --- | --- |
| 1 | scolios*.tw. | 490 |
| 2 | (brac* or corset* or orthos* or garment* or undergarment* or velcro or band* or belt* or fabric* or textile* or vest or vests or t shirt* or suit* or wearable* or cloth* or patch or stretchable*).tw. | 123605 |
| 3 | microcomputers/ or smartphones/ | 4948 |
| 4 | telemedicine/ | 9238 |
| 5 | mobile applications/ or mobile health/ | 5048 |
| 6 | (sensor or sensors or electronic* monitoring or digital* monitoring or scolios* track* or mulitplexed sensing or microfluidic device*).tw. | 9115 |
| 7 | (scolios* adj3 application*).tw. | 0 |
| 8 | (mobile application* or app or apps or mapp or eapp or microcomputer* or micro computer* or minicomputer* or mini computer* or smartphone* or smart phone* or handheld device*).tw. | 23106 |
| 9 | (mhealth or m health or mobile health or ehealth or e health or electronic* health or telemedicine or tele medicine or telehealth or tele health).tw. | 16749 |
| 10 | human robot* interfac*.tw. | 24 |
| 11 | ((soft or dynamic* or real time or remote or continous* or activity) adj2 (sensor* or sensing or device* or electronic* or technolog* or computer* or instrument* or system* or monitor* or robot* or track*)).tw. | 20413 |
| 12 | or/3-11 | 68310 |
| 13 | 2 and 12 | 4544 |
| 14 | wearable devices/ | 1029 |
| 15 | (e textile* or etextile* or electronic skin* or eskin* or e skin*).tw. | 91 |
| 16 | ((intelligen* or electro* or smart or flexib* or digital* or sensor* or sensing or technolog* or monitor* or software* or soft ware* or robot* or biosensor* or biosignal* or biomarker* or biometrics* or biomechanic* or active or soft*) adj5 (brac* or corset* or orthos* or garment* or undergarment* or velcro or band* or belt* or fabric* or textile* or vest or vests or t shirt* or suit* or wearable* or cloth* or patch or material* or stretchable*)).tw. | 8996 |
| 17 | ((wearable* or body* or posture) adj2 (sensor* or sensing or device* or electronic* or technolog* or computer* or instrument* or system* or monitor* or robot* or track*)).tw. | 4323 |
| 18 | or/13-17 | 14881 |
| 19 | 18 and 1 | 8 |

**Database**:	Cochrane via Wiley

**Date**:	21.05.2024

**Limits:**	-

**Results**:	71

| ID | Search | Hits |
| --- | --- | --- |
| #1 | [mh ^Scoliosis] | 736 |
| #2 | scolios*:ti,ab,kw | 1757 |
| #3 | #1 or #2 | 1757 |
| #4 | [mh ^"orthotic devices"] or [mh ^braces] | 1300 |
| #5 | (brac* or corset* or orthos* or garment* or undergarment* or velcro or band* or belt* or fabric* or textile* or vest or vests or t NEXT shirt* or suit* or wearable* or cloth* or patch or stretchable*):ti,ab,kw | 81392 |
| #6 | #4 or #5 | 81658 |
| #7 | [mh ^"Mobile Applications"] | 1963 |
| #8 | [mh ^microcomputers] | 294 |
| #9 | [mh ^"computers, handheld"] | 406 |
| #10 | [mh ^smartphone] | 1152 |
| #11 | [mh ^minicomputers] | 10 |
| #12 | [mh ^Telemedicine] | 4025 |
| #13 | (sensor or sensors or electronic* NEXT monitoring or digital* NEXT monitoring or scolios* NEXT track* or "mulitplexed sensing" or microfluidic NEXT device*):ti,ab,kw | 9852 |
| #14 | (scolios* NEAR/3 application*):ti,ab,kw | 2 |
| #15 | (mobile NEXT application* or app or apps or mapp or eapp or microcomputer* or micro NEXT computer* or minicomputer* or mini NEXT computer* or smartphone* or smart NEXT phone* or handheld NEXT device*):ti,ab,kw | 21517 |
| #16 | (mhealth or m NEXT health or mobile NEXT health or ehealth or e NEXT health or electronic* NEXT health or telemedicine or tele NEXT medicine or telehealth or tele NEXT health):ti,ab,kw | 16464 |
| #17 | (human NEXT robot* NEXT interfac*):tI,ab,kw | 1 |
| #18 | ((soft or dynamic* or "real time" or remote or continuous* or activity) NEAR/2 (sensor* or sensing or device* or electronic* or technolog* or computer* or instrument* or system* or monitor* or robot* or track*)):ti,ab,kw | 10172 |
| #19 | #7 or #8 or #9 or #10 or #11 or #12 or #13 or #14 or #15 or #16 or #17 or #18 | 50309 |
| #20 | #6 and #19 | 4415 |
| #21 | [mh ^"Wearable Electronic Devices"] | 292 |
| #22 | (e NEXT textile* or etextile* or electronic NEXT skin* or eskin* or e NEXT skin*):ti,ab,kw | 70 |
| #23 | ((intelligen* or electro* or smart or flexib* or digital* or sensor* or sensing or technolog* or monitor* or software* or soft NEXT ware* or robot* or biosensor* or biosignal* or biomarker* or biometrics* or biomechanic* or active or soft*) NEAR/5 (brac* or corset* or orthos* or garment* or undergarment* or velcro or band* or belt* or fabric* or textile* or vest or vests or t NEXT shirt* or suit* or wearable* or cloth* or patch or material* or stretchable*)):ti,ab,kw | 7180 |
| #24 | ((wearable* or body* or posture) NEAR/2 (sensor* or sensing or device* or electronic* or technolog* or computer* or instrument* or system* or monitor* or robot* or track*)):ti,ab,kw | 6427 |
| #25 | #20 or #21 or #22 or #23 or #24 | 14887 |
| #26 | #3 and #25 | 71 |

**Database**: Cinahl EBSCOhost

**Date**:	22.05.2024

**Limits:**	-

**Results:**	238

| **#** | **Query** | **Results** |
| --- | --- | --- |
| S1 | (MH "Scoliosis") OR (MH "Scoliosis, Idiopathic, Adolescent") | 8,244 |
| S2 | TI scolios* OR AB scolios* | 9,326 |
| S3 | S1 OR S2 | 10,747 |
| S4 | (MH "Orthoses") OR (MH "Orthoses Design") OR (MH "Orthoses Fitting") | 7,859 |
| S5 | TI ( (brac* or corset* or orthos* or garment* or undergarment* or velcro or band* or belt* or fabric* or textile* or vest or vests or "t shirt*" or suit* or wearable* or cloth* or patch or stretchable*) ) OR AB ( (brac* or corset* or orthos* or garment* or undergarment* or velcro or band* or belt* or fabric* or textile* or vest or vests or "t shirt*" or suit* or wearable* or cloth* or patch or stretchable*) ) | 145,276 |
| S6 | S4 OR S5 | 148,799 |
| S7 | (MH "Mobile Applications") | 13,636 |
| S8 | (MH "Microcomputers") OR (MH "Minicomputers") | 1,532 |
| S9 | (MH "Computers, Hand-Held") OR (MH "Smartphone") | 9,108 |
| S10 | (MH "Telemedicine") OR (MH "Telehealth") | 31,650 |
| S11 | TI ( (sensor or sensors or "electronic* monitoring" or "digital* monitoring" or "scolios* track*" or "mulitplexed sensing" or "microfluidic device*") ) OR AB ( (sensor or sensors or "electronic* monitoring" or "digital* monitoring" or "scolios* track*" or "mulitplexed sensing" or "microfluidic device*") ) | 12,797 |
| S12 | TI (scolios* N2 application*) OR AB (scolios* N2 application*) | 21 |
| S13 | TI ( ("mobile application*" or app or apps or mapp or eapp or microcomputer* or "micro computer*" or minicomputer* or "mini computer*" or smartphone* or "smart phone*" or "handheld device*") ) OR AB ( ("mobile application*" or app or apps or mapp or eapp or microcomputer* or "micro computer*" or minicomputer* or "mini computer*" or smartphone* or "smart phone*" or "handheld device*") ) | 22,416 |
| S14 | TI ( (mhealth or "m health" or "mobile health" or ehealth or "e health" or "electronic* health" or telemedicine or "tele medicine" or telehealth or "tele health") ) OR AB ( (mhealth or "m health" or "mobile health" or ehealth or "e health" or "electronic* health" or telemedicine or "tele medicine" or telehealth or "tele health") ) | 40,248 |
| S15 | TI "human robot* interfac*" OR AB "human robot* interfac*" | 9 |
| S16 | TI ( ((soft or dynamic* or "real time" or remote or continous* or activity) N1 (sensor* or sensing or device* or electronic* or technolog* or computer* or instrument* or system* or monitor* or robot* or track*)) ) OR AB ( ((soft or dynamic* or "real time" or remote or continous* or activity) N1 (sensor* or sensing or device* or electronic* or technolog* or computer* or instrument* or system* or monitor* or robot* or track*)) ) | 20,191 |
| S17 | S7 OR S8 OR S9 OR S10 OR S11 OR S12 OR S13 OR S14 OR S15 OR S16 | 116,518 |
| S18 | S6 AND S17 | 5,870 |
| S19 | (MH "Wearable Sensors") | 3,755 |
| S20 | TI ( ("e textile*" or etextile* or "electronic skin*" or eskin* or "e skin*") ) OR AB ( ("e textile*" or etextile* or "electronic skin*" or eskin* or "e skin*") ) | 58 |
| S21 | TI ( ((intelligen* or electro* or smart or flexib* or digital* or sensor* or sensing or technolog* or monitor* or software* or "soft ware*" or robot* or biosensor* or biosignal* or biomarker* or biometrics* or biomechanic* or active or soft*) N4 (brac* or corset* or orthos* or garment* or undergarment* or velcro or band* or belt* or fabric* or textile* or vest or vests or "t shirt*" or suit* or wearable* or cloth* or patch or material* or stretchable*)) ) OR AB ( ((intelligen* or electro* or smart or flexib* or digital* or sensor* or sensing or technolog* or monitor* or software* or "soft ware*" or robot* or biosensor* or biosignal* or biomarker* or biometrics* or biomechanic* or active or soft*) N4 (brac* or corset* or orthos* or garment* or undergarment* or velcro or band* or belt* or fabric* or textile* or vest or vests or "t shirt*" or suit* or wearable* or cloth* or patch or material* or stretchable*)) ) | 13,883 |
| S22 | TI ( ((wearable* or body* or posture) N1 (sensor* or sensing or device* or electronic* or technolog* or computer* or instrument* or system* or monitor* or robot* or track*)) ) OR AB ( ((wearable* or body* or posture) N1 (sensor* or sensing or device* or electronic* or technolog* or computer* or instrument* or system* or monitor* or robot* or track*)) ) | 6,012 |
| S23 | S18 OR S19 OR S20 OR S21 OR S22 | 23,350 |
| S24 | S3 AND S23 | 238 |

**Database**: Web of Science Core Collection

**Date**:	22.05.2024

**Limits:**	-

**Results**: 348

| # | Search Query | Results |
| --- | --- | --- |
| 1 | TS=scolios* | 23241 |
| 2 | TS=(brac* or corset* or orthos* or garment* or undergarment* or velcro or band* or belt* or fabric* or textile* or vest or vests or "t shirt*" or suit* or wearable* or cloth* or patch or stretchable*) | 4064949 |
| 3 | TS=(sensor or sensors or "electronic* monitoring" or "digital* monitoring" or "scolios* track*" or "mulitplexed sensing" or "microfluidic device*") | 810102 |
| 4 | TS=(scolios* NEAR/2 application*) | 39 |
| 5 | TS=("mobile application*" or app or apps or mapp or eapp or microcomputer* or "micro computer*" or minicomputer* or "mini computer*" or smartphone* or "smart phone*" or "handheld device*") | 142327 |
| 6 | TS=(mhealth or "m health" or "mobile health" or ehealth or "e health" or "electronic* health" or telemedicine or "tele medicine" or telehealth or "tele health") | 104347 |
| 7 | TS="human robot* interfac*" | 309 |
| 8 | TS=((soft or dynamic* or "real time" or remote or continous* or activity) NEAR/1 (sensor* or sensing or device* or electronic* or technolog* or computer* or instrument* or system* or monitor* or robot* or track*)) | 469342 |
| 9 | #3 OR #4 OR #5 OR #6 OR #7 OR #8 | 1433952 |
| 10 | #9 AND #2 | 286344 |
| 11 | TS=("e textile*" or etextile* or "electronic skin*" or eskin* or "e skin*") | 5837 |
| 12 | TS=((intelligen* or electro* or smart or flexib* or digital* or sensor* or sensing or technolog* or monitor* or software* or "soft ware*" or robot* or biosensor* or biosignal* or biomarker* or biometrics* or biomechanic* or active or soft*) NEAR/4 (brac* or corset* or orthos* or garment* or undergarment* or velcro or band* or belt* or fabric* or textile* or vest or vests or "t shirt*" or suit* or wearable* or cloth* or patch or material* or stretchable*)) | 654421 |
| 13 | #10 OR #11 OR #12 | 834934 |
| 14 | #13 AND #1 | 348 |

**Database**:	Scopus

**Date**:	22.05.2024

**Limits:**	-

**Results**:	640

( ( ( ( TITLE-ABS-KEY ( sensor OR sensors OR "electronic* monitoring" OR "digital* monitoring" OR "scolios* track*" OR "mulitplexed sensing" OR "microfluidic device*" ) ) OR ( TITLE-ABS-KEY ( scolios* W/2 application* ) ) OR ( TITLE-ABS-KEY ( "mobile application*" OR app OR apps OR mapp OR eapp OR microcomputer* OR "micro computer*" OR minicomputer* OR "mini computer*" OR smartphone* OR "smart phone*" OR "handheld device*" ) ) OR ( TITLE-ABS-KEY ( mhealth OR "m health" OR "mobile health" OR ehealth OR "e health" OR "electronic* health" OR telemedicine OR "tele medicine" OR telehealth OR "tele health" ) ) OR ( TITLE-ABS-KEY ( "human robot* interfac*" ) ) OR ( TITLE-ABS-KEY ( ( soft OR dynamic* OR "real time" OR remote OR continuous* OR activity ) W/1 ( sensor* OR sensing OR device* OR electronic* OR technolog* OR computer* OR instrument* OR system* OR monitor* OR robot* OR track* ) ) ) ) AND ( TITLE-ABS-KEY ( brac* OR corset* OR orthos* OR garment* OR undergarment* OR velcro OR band* OR belt* OR fabric* OR textile* OR vest OR vests OR "t shirt*" OR suit* OR wearable* OR cloth* OR patch OR stretchable* ) ) ) OR ( TITLE-ABS-KEY ( "e textile*" OR etextile* OR "electronic skin*" OR eskin* OR "e skin*" ) ) OR ( TITLE-ABS-KEY ( ( intelligen* OR electro* OR smart OR flexib* OR digital* OR sensor* OR sensing OR technolog* OR monitor* OR software* OR "soft ware*" OR robot* OR biosensor* OR biosignal* OR biomarker* OR biometrics* OR biomechanic* OR active OR soft* ) W/4 ( brac* OR corset* OR orthos* OR garment* OR undergarment* OR velcro OR band* OR belt* OR fabric* OR textile* OR vest OR vests OR "t shirt*" OR suit* OR wearable* OR cloth* OR patch OR material* OR stretchable* ) ) ) ) AND ( TITLE-ABS-KEY ( scolios* ) )

Search history 2: scoliosis AND braces AND user experiences

**Database**:	Ovid MEDLINE(R) ALL <1946 to May 20, 2024>

**Date**:	21.05.2024

**Limits:**	-

**Results**:	531

| **#** | **Searches** | **Results** |
| --- | --- | --- |
| 1 | Scoliosis/ | 21453 |
| 2 | scolios*.tw,kw,kf. | 26760 |
| 3 | 1 or 2 | 30873 |
| 4 | Braces/ | 6038 |
| 5 | Orthotic Devices/ | 7022 |
| 6 | (brac* or corset* or orthos* or garment* or undergarment* or velcro or band* or belt* or fabric* or textile* or vest or vests or t shirt* or suit* or wearable* or cloth* or patch or stretchable*).tw,kw,kf. | 1436687 |
| 7 | 4 or 5 or 6 | 1442152 |
| 8 | 3 and 7 | 3607 |
| 9 | "Surveys and Questionnaires"/ | 580932 |
| 10 | patient satisfaction/ or patient preference/ | 101147 |
| 11 | qualitative research/ | 87902 |
| 12 | interview/ | 31015 |
| 13 | Focus Groups/ | 37264 |
| 14 | ((user* or patient* or client*) adj3 (experience* or satisf* or preference* or perception* or view*)).tw,kw,kf. | 329700 |
| 15 | experience*.ti. | 321633 |
| 16 | (qualitativ* or interview* or focus group* or questionnaire* or survey*).tw,kw,kf. | 2039616 |
| 17 | or/9-16 | 2690685 |
| 18 | 8 and 17 | 531 |

**Database**:	Embase <1974 to 2024 Week 20>

**Date**:	21.05.2024

**Limits:**	-

**Results**:	791

| **#** | **Searches** | **Results** |
| --- | --- | --- |
| 1 | idiopathic scoliosis/ or adolescent idiopathic scoliosis/ or scoliosis/ | 39876 |
| 2 | scolios*.tw,kw,kf. | 35056 |
| 3 | 1 or 2 | 44776 |
| 4 | spinal brace/ or boston brace/ or jewett brace/ or milwaukee brace/ or pelvic belt/ or thoracolumbosacral orthosis/ | 1257 |
| 5 | brace/ or posture correction brace/ or corset/ or lumbosacral corset/ | 10927 |
| 6 | orthosis/ | 8276 |
| 7 | (brac* or corset* or orthos* or garment* or undergarment* or velcro or band* or belt* or fabric* or textile* or vest or vests or t shirt* or suit* or wearable* or cloth* or patch or stretchable*).tw,kw,kf. | 1663876 |
| 8 | 4 or 5 or 6 or 7 | 1670997 |
| 9 | health survey/ or health care survey/ | 263193 |
| 10 | questionnaire/ | 949120 |
| 11 | patient satisfaction/ | 177167 |
| 12 | patient preference/ | 28298 |
| 13 | qualitative research/ | 127545 |
| 14 | structured interview/ or video interview/ or interview/ or semi structured interview/ or telephone interview/ or unstructured interview/ or audio interview/ | 357311 |
| 15 | ((user* or patient* or client*) adj3 (experience* or satisf* or preference* or perception* or view*)).tw,kw,kf. | 511342 |
| 16 | experience*.ti. | 427594 |
| 17 | (qualitativ* or interview* or focus group* or questionnaire* or survey*).tw,kw,kf. | 2655259 |
| 18 | 9 or 10 or 11 or 12 or 13 or 14 or 15 or 16 or 17 | 3655933 |
| 19 | 3 and 8 and 18 | 791 |
|  | | |

**Database**:	APA PsycInfo <1806 to May Week 3 2024> via Ovid

**Date**:	21.05.2024

**Limits:**	-

**Results**:	22

| **#** | **Searches** | **Results** |
| --- | --- | --- |
| 1 | scolios*.tw. | 490 |
| 2 | (brac* or corset* or orthos* or garment* or undergarment* or velcro or band* or belt* or fabric* or textile* or vest or vests or t shirt* or suit* or wearable* or cloth* or patch or stretchable*).tw. | 123605 |
| 3 | 1 and 2 | 62 |
| 4 | Surveys/ | 14404 |
| 5 | Questionnaires/ | 27087 |
| 6 | Client Satisfaction/ | 6734 |
| 7 | Preferences/ | 23461 |
| 8 | Qualitative Methods/ | 11562 |
| 9 | Interviews/ | 13164 |
| 10 | Focus Group/ | 862 |
| 11 | ((user* or patient* or client*) adj3 (experience* or satisf* or preference* or perception* or view*)).tw. | 70251 |
| 12 | experience*.ti. | 120100 |
| 13 | (qualitativ* or interview* or focus group* or questionnaire* or survey*).tw. | 1107370 |
| 14 | 4 or 5 or 6 or 7 or 8 or 9 or 10 or 11 or 12 or 13 | 1221124 |
| 15 | 3 and 14 | 22 |

**Database**:	Cochrane via Wiley

**Date**:	21.05.2024

**Limits:**	-

**Results**:	107

| ID | Search | Hits |
| --- | --- | --- |
| #1 | [mh ^Scoliosis] | 736 |
| #2 | scolios*:ti,ab,kw | 1757 |
| #3 | #1 or #2 | 1757 |
| #4 | [mh ^Braces] | 586 |
| #5 | [mh ^"Orthotic Devices"] | 742 |
| #6 | (brac* or corset* or orthos* or garment* or undergarment* or velcro or band* or belt* or fabric* or textile* or vest or vests or t NEXT shirt* or suit* or wearable* or cloth* or patch or stretchable*):ti,ab,kw | 81392 |
| #7 | #4 or #5 or #6 | 81658 |
| #8 | #3 and #7 | 425 |
| #9 | [mh ^"Surveys and Questionnaires"] | 36292 |
| #10 | [mh ^"patient satisfaction"] | 14401 |
| #11 | [mh ^"patient preference"] | 1202 |
| #12 | [mh ^"qualitative research"] | 2290 |
| #13 | [mh ^interview] | 1 |
| #14 | [mh ^"Focus Groups"] | 1052 |
| #15 | ((user* or patient* or client*) NEAR/3 (experience* or satisf* or preference* or perception* or view)):ti,ab,kw | 78787 |
| #16 | experience*:ti | 139304 |
| #17 | (qualitativ* or interview* or focus group* or questionnaire* or survey*):ti,ab,kw | 278965 |
| #18 | #9 or #10 or #11 or #12 or #13 or #14 or #15 or #16 or #17 | 407714 |
| #19 | #8 and #18 | 107 |

**Database**:	Cinahl EBSCOhost

**Date**:	22.05.2024

**Limits:**	-

**Results**:	280

| **#** | **Query** | **Results** |
| --- | --- | --- |
| S1 | (MH "Scoliosis") OR (MH "Scoliosis, Idiopathic, Adolescent") | 8,244 |
| S2 | TI scolios* OR AB scolios* | 9,326 |
| S3 | S1 OR S2 | 10,747 |
| S4 | (MH "Orthoses") OR (MH "Orthoses Fitting") OR (MH "Orthoses Design") | 7,859 |
| S5 | TI ( (brac* or corset* or orthos* or garment* or undergarment* or velcro or band* or belt* or fabric* or textile* or vest or vests or "t shirt*" or suit* or wearable* or cloth* or patch or stretchable*) ) OR AB ( (brac* or corset* or orthos* or garment* or undergarment* or velcro or band* or belt* or fabric* or textile* or vest or vests or "t shirt*" or suit* or wearable* or cloth* or patch or stretchable*) ) | 145,276 |
| S6 | S4 OR S5 | 148,799 |
| S7 | S3 AND S6 | 1,479 |
| S8 | (MH "Survey Research") OR (MH "Surveys") | 188,930 |
| S9 | (MH "Questionnaires") OR (MH "Open-Ended Questionnaires") OR (MH "Structured Questionnaires") | 505,250 |
| S10 | (MH "Qualitative Studies") | 154,730 |
| S11 | (MH "Interviews") OR (MH "Semi-Structured Interview") OR (MH "Structured Interview") OR (MH "Unstructured Interview") | 266,236 |
| S12 | (MH "Focus Groups") | 52,493 |
| S13 | TI ( ((user* or patient* or client*) N2 (experience* or satisf* or preference* or perception* or view*)) ) OR AB ( ((user* or patient* or client*) N2 (experience* or satisf* or preference* or perception* or view*)) ) | 122,657 |
| S14 | TI experience* | 133,374 |
| S15 | TI ( (qualitativ* or interview* or "focus group*" or questionnaire* or survey*) ) OR AB ( (qualitativ* or interview* or "focus group*" or questionnaire* or survey*) ) | 859,628 |
| S16 | S8 OR S9 OR S10 OR S11 OR S12 OR S13 OR S14 OR S15 | 1,327,410 |
| S17 | S7 AND S16 | 280 |

**Database**:	Web of Science Core Collection

**Date**:	22.05.2024

**Limits:**	-

**Results**:	450

| # | Search Query | Results |
| --- | --- | --- |
| 1 | TS=scolios* | 23241 |
| 2 | TS=(brac* or corset* or orthos* or garment* or undergarment* or velcro or band* or belt* or fabric* or textile* or vest or vests or "t shirt*" or suit* or wearable* or cloth* or patch or stretchable*) | 4064949 |
| 3 | #2 AND #1 | 2468 |
| 4 | TS=((user* or patient* or client*) NEAR/2 (experience* or satisf* or preference* or perception* or view*)) | 339855 |
| 5 | TI=experience* | 428950 |
| 6 | TS=(qualitativ* or interview* or "focus group*" or questionnaire* or survey*) | 3186460 |
| 7 | #4 OR #5 OR #6 | 3728908 |
| 8 | #7 AND #3 | 450 |

**Database**:	Scopus

**Date**:	22.05.2024

**Limits:**	-

**Results**:	818

( ( TITLE-ABS-KEY ( ( user* OR patient* OR client* ) W/2 ( experience* OR satisf* OR preference* OR perception* OR view* ) ) ) OR ( TITLE ( experience* ) ) OR ( TITLE-ABS-KEY ( qualitativ* OR interview* OR "focus group*" OR questionnaire* OR survey* ) ) ) AND ( ( TITLE-ABS-KEY ( brac* OR corset* OR orthos* OR garment* OR undergarment* OR velcro OR band* OR belt* OR fabric* OR textile* OR vest OR vests OR "t shirt*" OR suit* OR wearable* OR cloth* OR patch OR stretchable* ) ) AND ( TITLE-ABS-KEY ( scolios* ) ) )
